# Identification of colorectal cancer associated biomarkers: an integrated analysis of miRNA expression

**DOI:** 10.18632/aging.203556

**Published:** 2021-09-21

**Authors:** André Fonseca, Sara Ventura Ramalhete, André Mestre, Ricardo Pires das Neves, Ana Marreiros, Pedro Castelo-Branco, Vânia Palma Roberto

**Affiliations:** 1 Faculty of Medicine and Biomedical Sciences (FMCB), University of Algarve, Campus de Gambelas, Faro 8005-139, Portugal; 2 Algarve Biomedical Center Research Institute (ABC-RI), Faro 8005-139, Portugal; 3 CNC, Center for Neuroscience and Cell Biology, CIBB - Centre for Innovative Biomedicine and Biotechnology, University of Coimbra, Coimbra 3004-517, Portugal; 4 IIIUC-Institute of Interdisciplinary Research, University of Coimbra, Coimbra 3030-789, Portugal; 5 Champalimaud Research Program, Champalimaud Center for the Unknown, Lisbon 1400-038, Portugal; 6 Centre of Marine Sciences (CCMAR), University of Algarve, Faro 8005-139, Portugal

**Keywords:** microRNAs, biomarker, colorectal cancer, diagnosis, prognosis

## Abstract

Colorectal cancer is one of the leading causes of cancer-related deaths worldwide. This complex disease still holds severe problems concerning diagnosis due to the high invasiveness nature of colonoscopy and the low accuracy of the alternative diagnostic methods. Additionally, patient heterogeneity even within the same stage is not properly reflected in the current stratification system. This scenario highlights the need for new biomarkers to improve non-invasive screenings and clinical management of patients.

MicroRNAs (miRNAs) have emerged as good candidate biomarkers in cancer as they are stable molecules, easily measurable and detected in body fluids thus allowing for non-invasive diagnosis and/or prognosis.

In this study, we performed an integrated analysis first using 4 different datasets (discovery cohorts) to identify miRNAs associated with colorectal cancer development, unveil their role in this disease by identifying putative targets and regulatory networks and investigate their ability to serve as biomarkers. We have identified 26 differentially expressed miRNAs which interact with frequently deregulated genes known to participate in commonly altered pathways in colorectal cancer. Most of these miRNAs have high diagnostic power, and their prognostic potential is evidenced by panels of 5 miRNAs able to predict the outcome of stage II and III colorectal cancer patients. Notably, 8 miRNAs were validated in three additional independent cohorts (validation cohorts) including a plasma cohort thus reinforcing the value of miRNAs as non-invasive biomarkers.

## INTRODUCTION

Colorectal cancer (CRC) is the third most diagnosed cancer worldwide with over 1.84 million novel cases reported in 2018, preceded only by lung, and breast cancers [1, 2]. Responsible for more than 880.729 deaths (9.2% of all tumour deaths) CRC is the second deadliest cancer worldwide representing a major public health problem [1]. Approximately 30% more prominent in men than women, CRC incidence is generally low for individuals younger than 50 years old in both genders and strongly increases with age [3–5].

Currently, colonoscopy is the gold-standard test for CRC diagnosis however, due to its highly invasive nature, patients usually tend to avoid it [6–9]. This issue, associated with the fact that patients with CRC are often asymptomatic in the early stages of the disease, leads CRC to go undetected until later stages of disease when cancer has already grown or spread to other tissues [10]. As the disease progresses, patient survival rates significantly decline stressing that early identification of CRC is of particular importance [11, 12]. Therefore, in order to increase test-adherence more appealing techniques are in need. Alternative non-invasive tests, such as the faecal occult blood tests (FOBTs) and serum biomarkers as Carcinoembryonic antigen (CEA) and the Carbohydrate antigen 19-9 (CA19-9), have been proposed for CRC but, their lack of specificity and sensitivity has limited their use [3, 6, 8, 13–15]. Furthermore, CEA and CA19-9 usually only present elevated serum concentrations in cases of advanced CRC, thus limiting their diagnostic value in earlier cases [14, 15].

Accurate assessment of CRC patient prognosis and optimal treatment selection is also challenging and mainly based on histological characteristics and pathological staging [16]. According to these features, patients are stratified into one of the tumour–node–metastasis (TNM) stages that range from I (the less advanced stage of the disease) to IV (the most advanced stage), and treatment is then selected accordingly [5, 17, 18]. Nevertheless, even after stratification, optimal individual therapy and survival times are very heterogeneous for patients within the same stage, significantly hampering a quality assessment of prognosis [16]. This scenario highlights the necessity for a more precise clinical management system for CRC, especially between stages II and III where it's most difficult to properly discern patients and thus to provide accurate treatment [19]. In this sense, the identification of novel, more efficient and less invasive biomarkers may help to improve the effectiveness of both diagnosis and prognosis of CRC patients.

As a complex disease, CRC arises from the progressive accumulation of both genetic and epigenetic alterations over the course of 10 to 20 years that ultimately result in the transformation of the normal glandular epithelium into early and then advance cancer [20–22]. Some of the most known specific genetic alterations in CRC are mutations in key genes that deregulate several cellular homeostatic functions from proliferation, differentiation, adhesion, migration, and cell death to DNA stability and repair [16, 23]. Mutations occurring in the genes *APC, TP53, KRAS, TGFβ* and *PIK3CA* are some of the most observed in CRC and have often been proposed as genetic biomarkers [23, 24]. Epigenetic modifications, such as DNA methylation, post-translational histone modifications and microRNAs post-transcriptional regulation also have a key role in CRC development and complement the molecular landscape of this disease [25, 26]. Epigenetics broadly consists in regulating gene expression without altering the DNA sequence and occurs in normal tissues, being of extreme importance during embryonic development, imprinting and tissue differentiation [25]. Nevertheless, when disturbed, epigenetic mechanisms can alter cellular homeostasis favouring tumour development [25–27].

Since the discovery of microRNAs (miRNAs) in patients with chronic lymphocytic leukaemia in 2002, their role in regulating post-transcriptional gene expression in cancer development, progression and metastasis has been well-established in the field [28–37]. MiRNAs are small non-coding RNAs, of approximately 20-22 nucleotides, that are processed from larger transcripts and exert a fine-tuning regulation by binding to the 3’-untranslated region (UTR) of their targets messenger RNA (mRNA) in the cytoplasm [25, 38, 39]. One single miRNA can regulate hundreds of target genes primarily by inducing translational repression, but they can also lead to mRNA cleavage and consequent decay [38, 39]. In both situations, the interaction of miRNAs with their target mRNAs results in a lower expression of the protein levels [38, 39]. They are highly stable molecules, with cell and tissue relative high specificity and easily measurable in different biological samples from tissues to saliva, serum and faeces [38, 40–43]. Due to their features, miRNAs have emerged as good non-invasive biomarker candidates for the detection of pre-cancerous/cancerous lesions as well as therapeutic targets [41–47]. Of notice, technological advances allowing large-scale miRNA profiling and miRNA regulatory network analysis have been crucial to understanding the role of miRNAs in pathological contexts [48–50]. Accordingly, several therapeutical strategies relying on mimics and antimiRs are currently in clinical trials as is the case of MRX34 (miR-34 mimic), in a study for the treatment of patients with advanced solid tumours including CRC [41, 51]. Although several miRNAs have been reported as promising cancer biomarkers [37, 42, 43, 45, 52–57] only a few are available to clinicians as the miRNA panel ThyraMIR® (Interpace Biosciences, Inc.®) for thyroid cancer diagnosis. Regarding CRC, despite the advances in the field and the number of miRNAs identified as potential biomarkers, their progress to the clinic has been compromised in part by contradictory results of independent studies and lack of consistency between biomarker panels [13, 58].

In this sense, here we propose to identify and scrutinize panels of miRNAs with the ability to behave as diagnostic and/or prognostic tools for CRC by compiling data from different studies to assure a bona fide data analysis and more concise results. This approach revealed novel miRNA panels able to accurately distinguish malignant from normal tissue, with high sensitivity and specificity, and to predict patient outcome, particularly in stages II and III. Our findings highlight promising candidate biomarkers to improve colorectal cancer clinical management.

## RESULTS

MicroRNAs are promising candidates for CRC screening and management. Since one biomarker is not enough to diagnose disease, a group of markers should increase sensitivity and specificity [59–61]. In that sense, here we aim to discover miRNAs associated with CRC development and/or progression and evaluate their combined diagnostic and prognostic values as panels of miRNAs.

### MiRNA expression dataset selection

**Figure 1:**
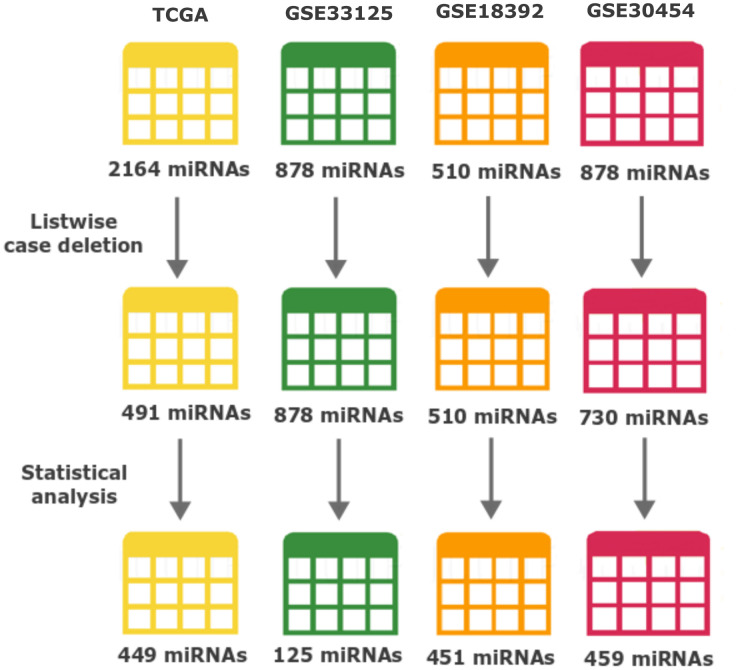
**Number of miRNAs throughout data processing and statistical analysis.** The number of miRNAs after each major event (Listwise case deletion and Statistical analysis) is shown below each dataset. Different colours represent: yellow TCGA, green GSE33215, orange GSE18392 and reddish-purple GSE30454. From the top, the numbers of miRNAs represent: the initially number of miRNAs present in each dataset before any processing step; the number of miRNAs after removing the ones with more than 50% information missing (modified listwise case deletion); and the number of miRNAs after performing all the statistics and used to perform the identification of differently expressed miRNAs across datasets.

In order to perform an integrated analysis, we combined data from 4 datasets (discovery cohorts), obtained through Illumina platforms: the TCGA colon and cancer cohorts (TCGA-COAD/READ, from now on here referred as the TCGA cohort or dataset) and 3 GEO datasets that fitted our inclusion criteria (Table 1). TCGA is the most complete dataset used in our study, with numerous multi-omics data and patient clinical history and therefore was the dataset used for the prognostic analysis. The TCGA also comprised the largest number of miRNAs with a total number of 2164, followed by GSE33125 and GSE30454 with 878 each and GSE18392 with 510 miRNAs (Table 1 and Figure 1). In total, our bioinformatics analyses were carried out on 588 samples of which 69 were normal tissue and 519 tumour samples of colorectal carcinoma. Table 1 summarizes a detailed description of the discovery 4 datasets.

**Table 1:** Detailed patient information for colorectal cancer datasets used in miRNA expression analysis.

**Datasets**	**TCGA**	**GSE33125**	**GSE18392**	**GSE30454**
Number of miRNAs available	2164	878	510	878
Patient nationality	USA	Italy	USA	Spain
Platform	Illumina HiSeq miRNA Seq	Illumina Human v2 MicroRNA expression beadchip	Illumina Human v1 MicroRNA expression beadchip	Illumina Human v2 MicroRNA expression beadchip
Tissue type	Tumour and normal tissue	Paired tumour and normal tissue	Tumour and healthy tissue	Tumour and healthy tissue
**Number of samples**				
Tumour	340 (97%)	9 (50%)	116 (80%)	20 (27%)
Normal	11 (3%)	9 (50%)	29 (20%)	54 (73%)
**Age (mean ± SD^1^ years)**				
Tumour sample patients	65 ± 13	-	-	59 ± 16
Normal sample patients	68 ± 19	-	-	64 ± 16
**Gender**				
Female	163 (46%)	-	-	43 (58%)
Male	185 (53%)	-	-	31 (42%)
Unknown	3 (1%)	18 (100%)	145 (100%)	-
**Tumour Site**				
Colon	259 (74%)	18 (100%)	145 (100%)	74 (100%)
Rectum	92 (26%)	-	-	-

1 – standard deviation.

“-“– inexistent or unspecified.

### Identification of differently expressed miRNAs in CRC

**Figure 2:**
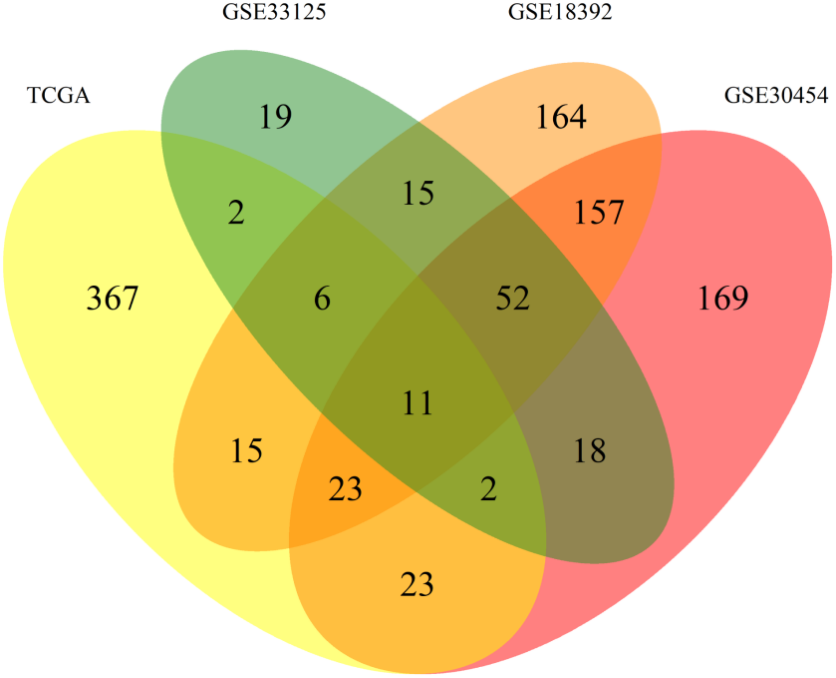
**Venn diagram of the differently expressed miRNAs between datasets.** Allocation of the 1043 differently expressed miRNAs found between the 4 datasets used in this work. Each dataset is represented by a colour, TCGA (yellow), GSE30545 (reddish purple), GSE33215 (green) and GSE18392 (orange). The number in each overlay of datasets represents the common miRNAs between those datasets.

To normalize our analysis, the 3 datasets from GEO were transformed and expressed as log_2_ values to exhibit the same scale value as the TCGA dataset. From there all datasets were processed equally. We started by applying listwise deletion and outlier removal followed by a process of statistical analysis. All steps of this analysis resulted in the elimination of miRNAs as resumed in Figure 1. Proportionally, the GSE33125 dataset reported the biggest miRNA loss, with almost 86% of all miRNAs being excluded, and a total of 125 miRNAs remaining for further analysis. GSE30454, GSE18392 and TCGA exhibited losses close to 37%, 12% and 9% respectively, totalizing 459, 451 and 449 miRNAs remaining in each dataset (Figure 1). We then proceeded to identify which miRNAs were present in at least 2 of the 3 GEO datasets while simultaneously being present in the TCGA. A total of 42 miRNAs fulfilled our criteria. From these, 31 miRNAs were simultaneously found in 3 datasets: 6 in TCGA, GSE33125 and GSE18392, 23 in TCGA, GSE30454 and GSE18392 and 2 in TCGA, GSE30454 and GSE33125 (Figure 2). Notably, 11 miRNAs were found in all 4 datasets at the same time (Figure 2).

Finally, we calculated the log_2_FC between tumour and normal tissue samples for the identified 42 miRNAs. Since this study only involves human samples, the name of each miRNA from now on will not include the prefix related to the species (hsa-).

**Figure 3:**
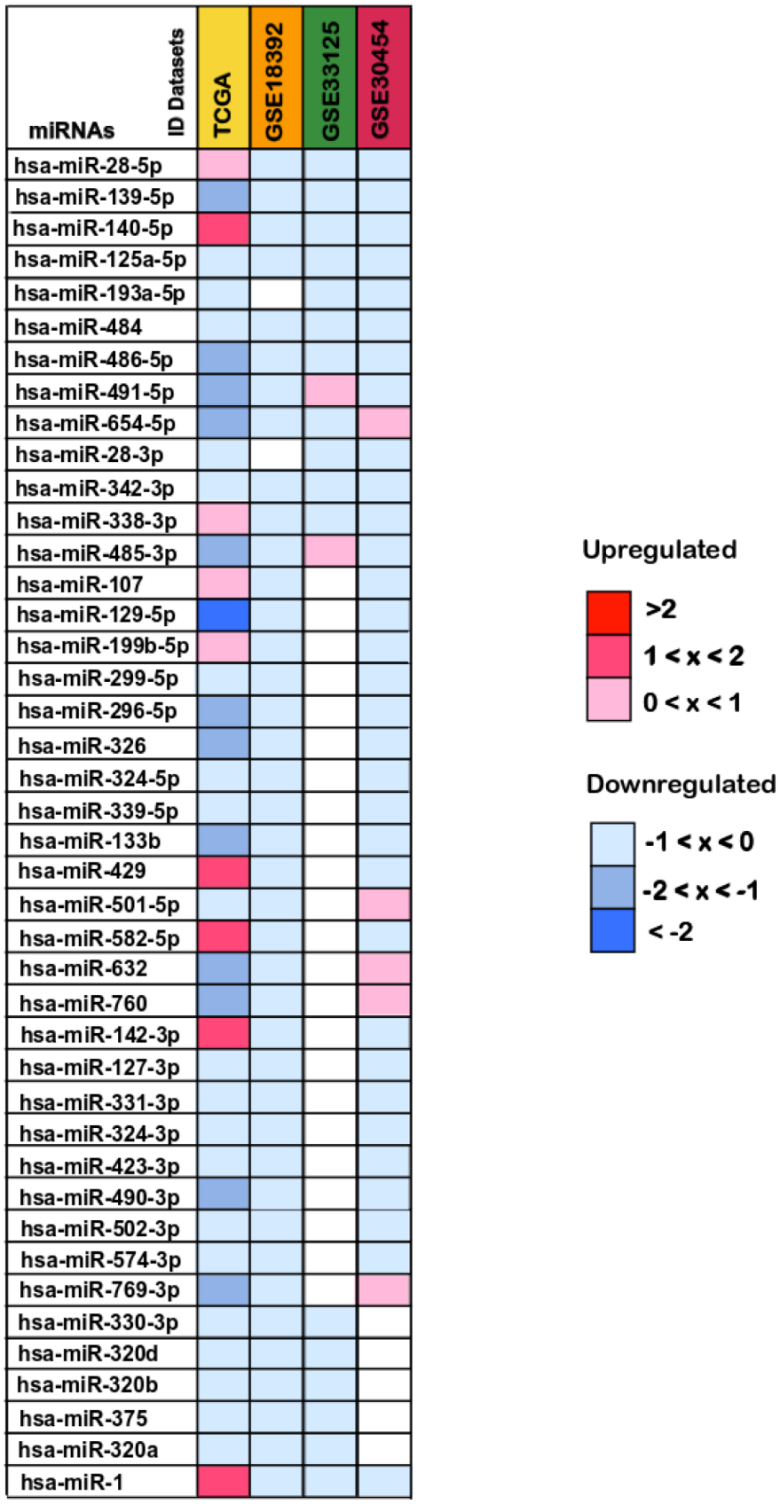
**Differentially expressed miRNAs between colorectal cancer and normal tissue samples.** The log_2_(FC) values calculated for each dataset are reported with red scale boxes for upregulated miRNAs and blue scale boxes for the downregulated miRNAs. White boxes represent the inexistence of the miRNA on the dataset. Only the miRNAs differentially expressed in at least 2 datasets while simultaneously being present in the TCGA dataset are displayed.

From these, 26 miRNAs presented lower expression levels in colorectal cancer tissues when compared to normal tissues and exhibited the same expression pattern in all the datasets where they were present: miR-139-5p, miR-125a-5p, miR-193a-5p, miR-484, miR-486-5p, miR-28-3p, miR-342-3p, miR-129-5p, miR-299-5p, miR-296-5p, miR-326, miR-324-5p, miR-339-5p, miR-133b, miR-127-3p, miR-331-3p, miR-324-3p, miR-423-3p, miR-490-3p, miR-502-3p, miR-574-3p, miR-330-3p, miR-320d, miR-320b, miR-375 and miR-320a (Figure 3). The remaining 16 miRNAs (miR-491-5p, miR-654-5p, miR-485-3p, miR-632, miR-76, miR-769-3p, miR-28-5p, miR-140-5p, miR-338-3p, miR-107, miR-199b-5p, miR-429, miR-501-5p, miR-582-5p, miR-142-3p and miR-1), exhibited opposite expression values in at least one dataset in which they were present and thus no regulation pattern could be perceived (Figure 3). Therefore, these miRNAs were excluded from the subsequent analyses.

Our results also point out different magnitudes of the log_2_(FC) values across datasets. While the TCGA dataset log_2_(FC) values ranged from less than -2 to almost 2, for downregulated and upregulated miRNAs respectively, in the remaining datasets the log_2_(FC) values were never below -1 or higher than 1 (Figure 3). Interestingly, the miRNA with the highest deregulation pattern overall (miR-129-5p) and the most downregulated in TCGA with a log_2_(FC) value of -2, is reported as a frequently downregulated miRNA in CRC, supporting our findings [62, 63].

### Gene target analysis of selected miRNAs

To better understand the role of the 26 downregulated miRNAs in CRC, we used the MirTarBase to identify functional miRNA-target interactions (MTIs). Our analysis identified a total of 309 different human target genes previously experimentally validated (either by report assay, western blot or qPCR) in a total of 384 papers (Supplementary Table 1). Among the target genes identified, *ERBB2* was the most prevalent, described in 12 different papers as regulated by miR-125a-5p, miR-331-3p, miR-375 and miR-193a-5p. *MCL1* was the second most frequent gene being reported in 9 papers as modulated by miR-133b, miR-320a, miR-139-5p and miR-125a-5p. *IGF1R* and *SP1* genes followed, both cited in 8 occasions (Supplementary Table 1).

**Figure 4:**
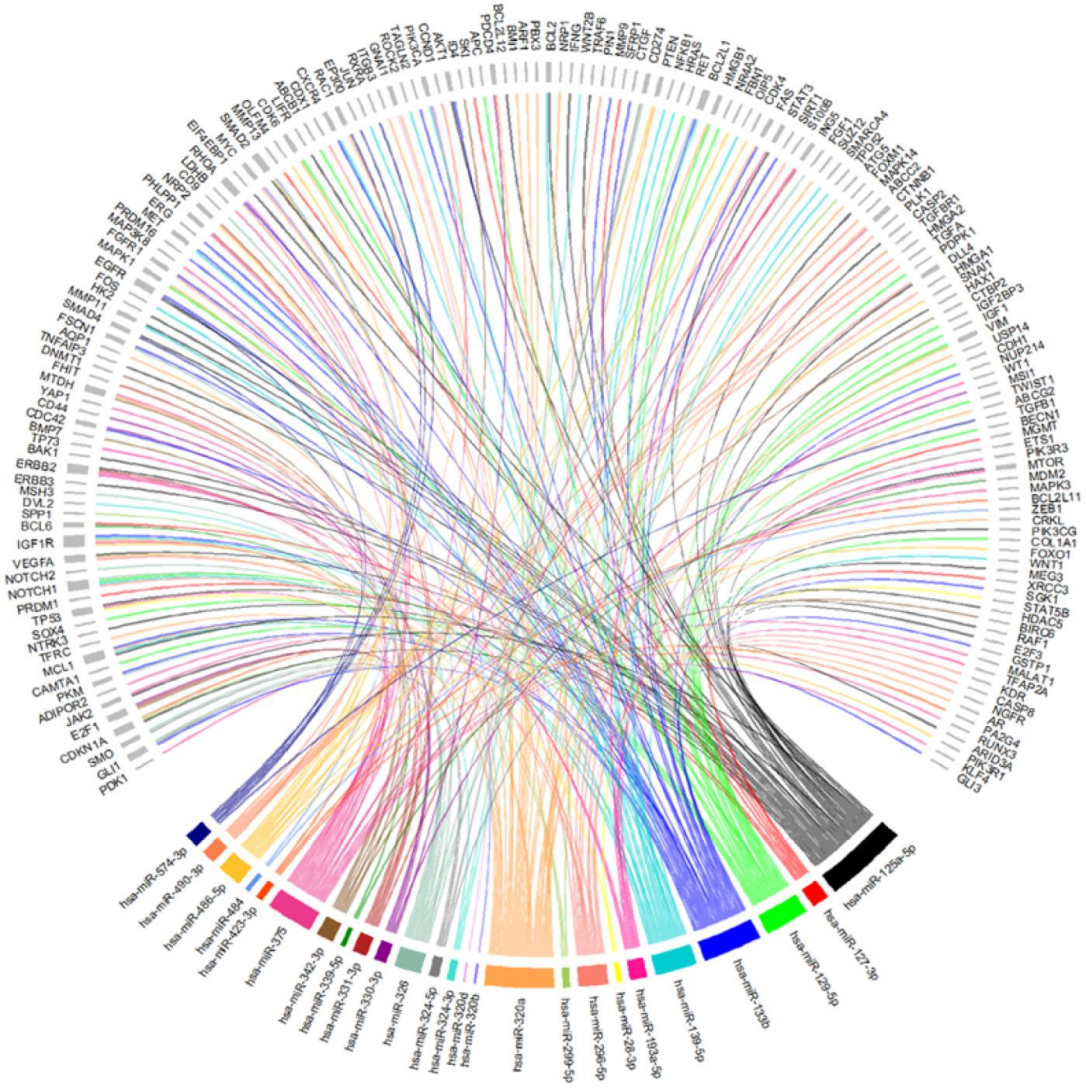
**Chord-dendrogram of the interactions between differently expressed miRNAs and altered genes in CRC.** The interactions between the 25 differently expressed miRNAs identified and the 173 experimentally validated target genes were obtained through MirTarBase. Target genes’ role in CRC is according to CoReCG database. All targets are represented in grey, while each miRNA is represented by one colour. MiRNA:target interactions are represented by a line of the same colour of the respective miRNA. The size of rectangle next to the name of the miRNAs and target genes is proportional to the number of interactions they perform.

Then, to understand which of the 26 miRNAs could be playing a role in the development of CRC, the 309 targets identified in the MTIs were screened in the CoReCG database which highlighted 173 genes previously associated to CRC (Figure 4 and Supplementary Figure 1 and Supplementary Table 2). From our set of 26 miRNAs, all were found to interact with at least one gene involved in CRC except for miR-502-3p. Among the remaining 25 miRNAs, miR-125a-5p a well-known deregulated miRNA in CRC [37] presented the highest number of interactions with CRC associated genes, interacting with a total of 44 genes among which were *AKT1*, *EGFR*, *TP53*, *VEGFA*, *SMAD2* and *SMAD4* (Figure 4 and Supplementary Table 2). MiR-133b which has also been reported as showing an altered expression in CRC [57], interacted with 38 genes including *AKT1*, *EGFR*, *MET*, *FGFR1* among other frequently altered genes in CRC. MiR-320a showed a total of 35 interactions, proceeded by miR-375 with 34, miR-139-5p with 24 and miR-129-5p with 22 interactions. Altogether these miRNAs were the ones with the highest number of interactions with commonly altered genes in CRC suggesting their potential contribution to the development of this disease (Figure 4, Supplementary Figure 1 and Supplementary Table 2). Besides the genes above mentioned other genes such as *APC*, *PIK3CA*, *PTEN*, *RUNX3* and *CTNNB1*, are some additional examples of the well-known altered genes in CRC targeted by some of the 25 miRNAs identified [25, 64–69] (Figure 4 and Supplementary Table 2). MiR-125a-5p, miR-375 and miR-28-3p were found to interact with *TP53*. Simultaneously, miR-125a-5p was also found to regulate both *SMAD2* and *SMAD4*. *SMAD4* was also a target gene of miR-574-3p. *PTEN* was a target of miR-486-5p and miR-320a, which also regulates *CTNNB1*. MiR-375, besides interacting with *TP53* also regulates *CTNBB1*, *RUNX3* and *PIK3CA*. *PIK3CA* was also found to be targeted by miR-139-5p. Finally, our results demonstrate that *APC* is a target of miR-129-5p (Figure 4, Supplementary Figure 1 and Supplementary Table 2). These results suggest that miRNAs may play a role in the development or progression of CRC through the regulation of key genes found frequently altered or mutated in CRC.

**Figure 5:**
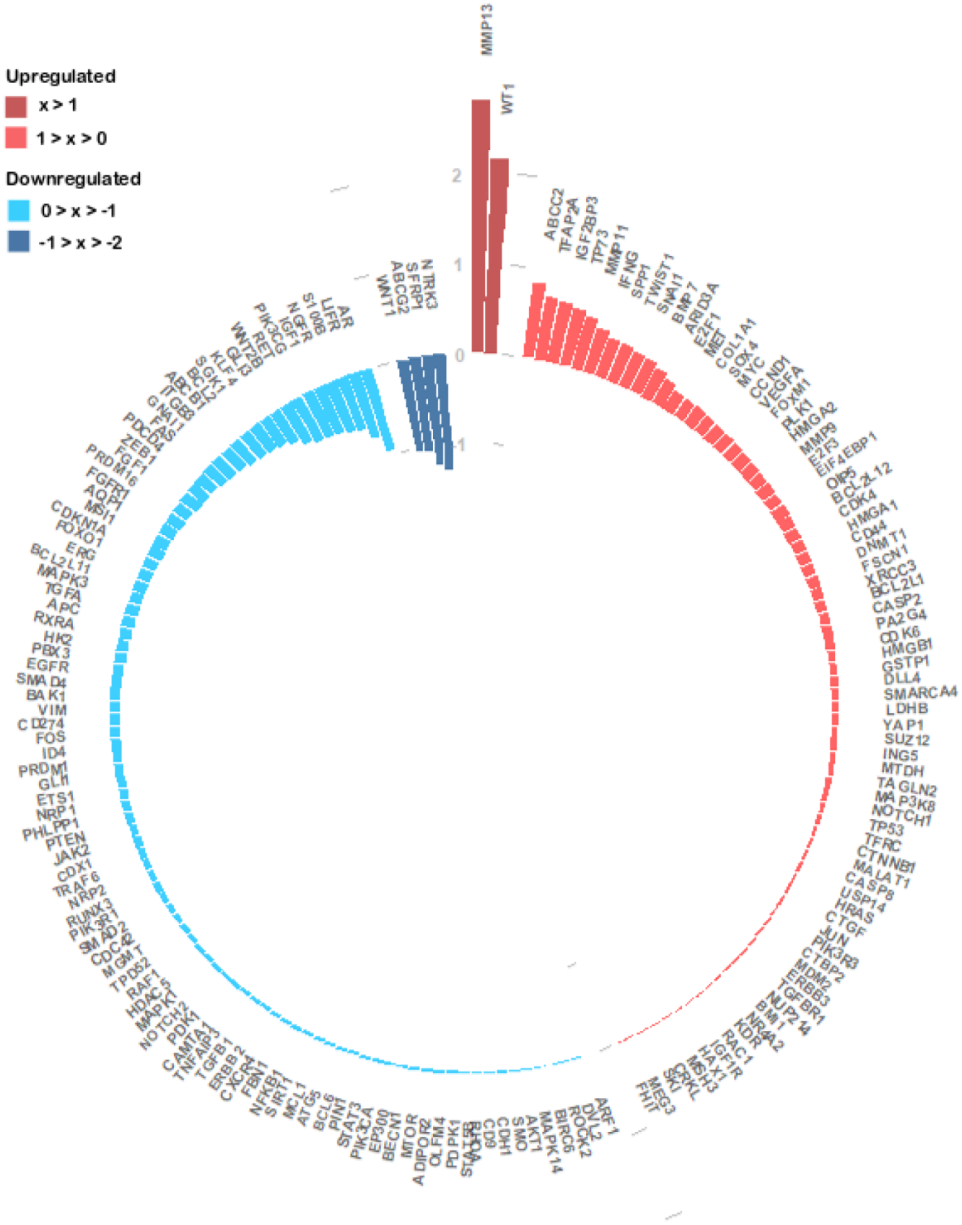
**Circular barplot evidencing the expression of the 172 experimentally validated miRNA target genes.** The expression values of the 172 genes were obtained from the TCGA colon and rectal cohorts and the log_2_(FC) values between the primary tumour and normal tissue samples were calculated. The log_2_(FC) value of each gene is given by the length and tone of each coloured bar in accordance with each gene regulation status (red colours – upregulated; blue colours- downregulated).

To get more insights into the miRNA regulation in the specific context of CRC, we investigated the 173 genes expression patterns and its association with the 25 miRNAs expression profile, in the TCGA dataset. We analysed 172 genes since information for PKM was missing. We found 76 upregulated genes and 96 downregulated genes in tumour samples (Figure 5 and Supplementary Table 3). *NTRK3*, *SFRP1*, *ABCG2* and *WNT1* with log_2_(FC) values of approximately -1.28, -1.22, -1.05 and -1.03 respectively (Figure 5) were the genes with the lowest expression values. Oppositely *MMP13* (log_2_(FC) ≈ 2.80) and *WT1* (log_2_(FC) ≈ 2.16) were the most upregulated genes followed by *ABCC2* (log_2_(FC) ≈ 0.85) and *TFAP2A* (log_2_(FC) ≈ 0.71) (Figure 5 and Supplementary Table 3). Correlation analysis between the expression of the 25 miRNA expression levels and their targets revealed that 104 miRNA-target genes interactions were statistically significant. From the 42 negative correlations, 10 had a *p-value* < 0.0001 while for the 62 positive interactions a *p-value* < 0.0001 was found in 25 associations. These 35 associations also presented the highest correlation coefficients, ranging from 0.215 to 0.478 in absolute value (Supplementary Table 4). The strongest negative interaction identified was miR-375:*YAP1* (correlation coefficient = -0.478; *p-value* = 4.99x10^-20^), followed by miR-484:*ZEB1* (correlation coefficient = -0.381; *p-value* = 1.17x10^-12^) and by miR-375:*MAP3K8* (correlation coefficient = -0.307; *p-value* = 1.17x10^-8^). On the other hand, the strongest positive interaction was identified between miR-375:*KLF4* (correlation coefficient = 0.427; *p-value* = 7.62x10^-16^), followed by the interactions miR-133b:GLI3 (correlation coefficient = 0,417; *p-value* = 5.22x10^-14^) and miR-133b:*FGFR1* (correlation coefficient = 0,386; *p-value* = 4.42x10^-12^). MiR-133 also interacted with *ERG* (correlation coefficient = 0.366; *p-value* = 6.89x10^-11^) and *GLI1* (correlation coefficient = 0.311; *p-value* = 4.14x10^-8^). MiR-125a-5p which we found to target the highest number of CRC altered genes, displayed interactions with *MEG3* (correlation coefficient = 0.373; *p-value* = 5.86x10^-12^) and *HDAC5* (correlation coefficient = 0.302; *p-value* = 3.87x10^-8^). MiR-129-5p presented an interaction with *IGF1* (correlation coefficient = 0.312; *p-value* = 1.70x10^-8^) (Supplementary Table 4).

Altogether, these results suggest that miR-125a-5p, miR-133b, miR-375 and miR-129-5p, which were among the miRNAs with highest number of CRC-related targets, may play a key role in the altered gene expression observed in CRC.

### Pathway analysis of the miRNAs target genes

To further understand the role of the 25 differentially expressed miRNAs in CRC development we investigated the molecular mechanisms of their 173 target genes. An enrichment pathway analysis using KEEG at DAVID database revealed a total of 104 pathways (*p-value* ≤ 0.05, Supplementary Table 5). The results pointed to the key role of miRNAs in cancer since pathways such as “Pathways in Cancer” came up on the top of our list (*p-value =* 6.44x10^-49^; FDR of 7.98x10^-46^), followed by “Prostate cancer” in the 3^rd^ position (*p-value* = 1.53x10^-28^; FDR = 1.9x10^-25^), “Pancreatic cancer” in the 4^th^ position (*p-value* = 3.2x10^-28^; FDR = 4.0x10^-25^) and “MicroRNAs in Cancer” in the 8^th^ position (*p-value* = 9.3x10^-23^; FDR 1.2x10^-19^) (Supplementary Table 5), evidenced the enrolment of these miRNAs in cancer. The “Colorectal cancer” pathway (*p-value =* 1.4x10^-22^; FDR =1.7x10^-19^, Supplementary Table 5) was in the 10^th^ position, stressing that the 25 miRNAs might play a role in CRC.

Furthermore, several pathways usually found altered in CRC came up in our analysis such as “PI3K-Akt signalling pathway” (*p-value* = 1.2x10^-21^; FDR = 1.9x10^-20^), “RAS signalling pathway” (*p-value* = 8.1x10^-11^; FDR = 4.5x10^-10^), “TP53 signalling pathway” (*p-value* = 7.1x10^-7^; FDR = 2.3x10^-6^), “TGF-beta signalling pathway” (*p-value* = 7.6x10^-7^; FDR = 2.4X10^-6^), “WNT signalling pathway” (*p-value* = 8.5x10^-8^ and FDR = 3.0x10^-7^ and “MAPK signalling pathway*” (p-value* = 2.39x10^-08^; FDR = 2.96x10^-05^). Additionally, cellular functions often aberrant in colorectal cancer such as “Apoptosis” (*p-value* = 3.4x10^-7^; FDR = 1.2x10^-6^) and “Cell cycle” (*p-value* = 9.9x10^-7^; FDR = 3.0x10^-6^) were found in our analysis (Supplementary Table 5). Together these results support the association between the 25 deregulated miRNAs and several alterations often reported in CRC.

**Figure 6:**
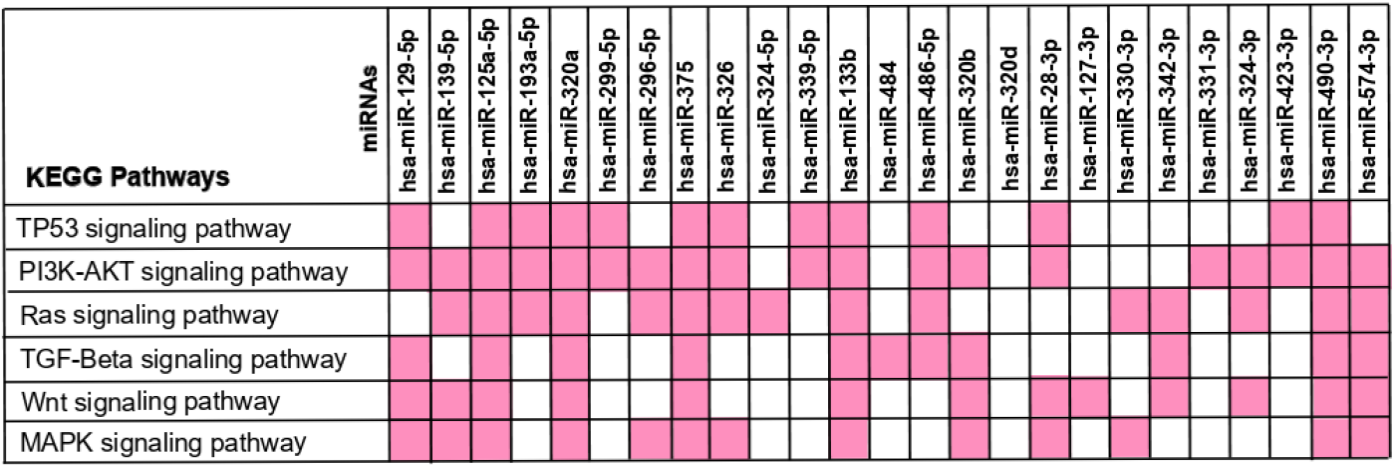
**KEGG pathway analysis – interaction between the 25 differently expressed miRNAs and the frequently altered pathways in colorectal cancer.** The 173 miRNA-target genes were used to perform the enrichment pathway analysis using KEEG at DAVID database. The miRNA-target genes found to be involved in CRC were crossed with the genes intervening in each signalling pathway in order to identify the miRNAs affecting each pathway. The miRNAs interaction with each KEGG Pathway is reported in pink.

We then aimed to understand in which frequently altered pathways in CRC (TP53, PI3K-AKT, RAS, TGF-Beta, WNT and MAPK signalling pathways) each miRNA was intervening [70–75]. For that, we crossed the miRNA-target genes found to be involved in CRC with the genes intervening in each of the signalling pathways. Our results show that most of the miRNAs were involved in at least one of these pathways, except for miR-320d (Figure 6). More interestingly, we found that several miRNAs seem to intervene in more than one pathway, and 5 miRNAs (miR-125a-5p, miR-320a, miR-375, miR-133b and miR-490-3p) interact with all the six pathways. Not surprisingly, apart from miR-490-3p these miRNAs represent the ones targeting the highest number of genes involved in CRC. Therefore, by targeting multiple genes that are constituents in these pathways it’s plausible that altered expression of these miRNAs may contribute to the aberration of frequently altered pathways in CRC (Figures 4–6). These results support the hypothesis that aberrant miRNA expression may contribute for the occurrence of several alteration events observed during CRC.

### Identification of potential diagnostic biomarkers for colorectal cancer

**Figure 7:**
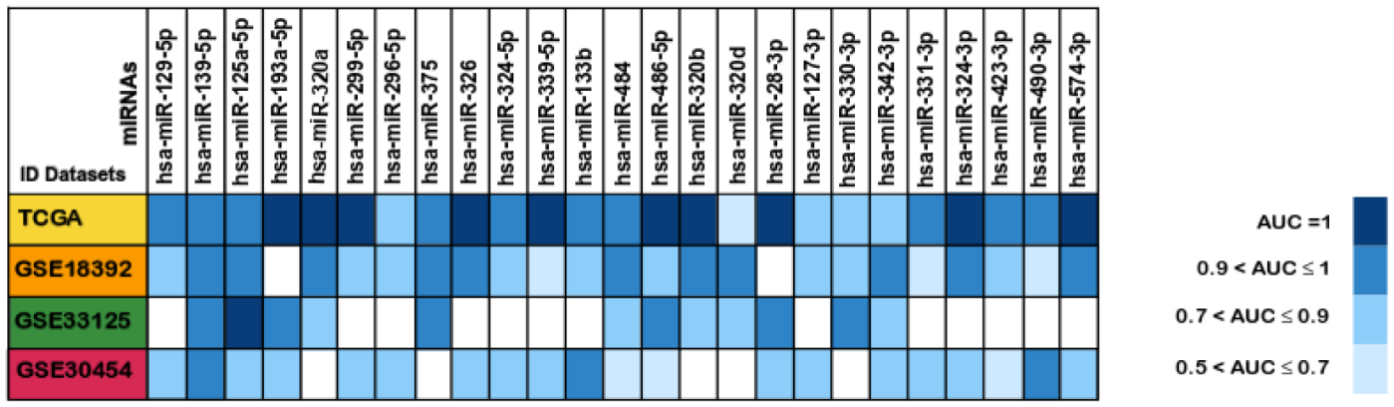
**Areas under the ROC curve (AUC) of the 25 differentially expressed miRNAs between colorectal cancer and normal tissues samples.** The miRNAs AUC values in each of the datasets TCGA (Yellow), GSE18392 (orange), GSE33125 (Green) and GSE30454 (reddish purple) are reported as blue scale boxes. MiRNAs with AUC = 1 were considered perfect diagnostic biomarkers, 0.9 < AUC < 1 highly accurate, 0.7 < AUC ≤ 0.9 moderately accurate and 0.5 < AUC ≤ 0.7 less accurate [76].

Given the potential key role in CRC of the 25 miRNAs identified in this study, we then investigated their diagnostic value. For that, Receiver Operating Characteristic (ROC) curve analysis was performed in all the datasets for the 25 differently expressed miRNAs and the Area Under the Curve (AUC) values were obtained in order to determine their ability to distinguish between colorectal cancer tissue and normal tissue. The diagnostic value of each miRNA was here characterized according to the classification by Greiner and colleagues as: less accurate (0.5 < AUC ≤ 0.7), moderately accurate (0.7 < AUC ≤ 0.9), highly accurate (0.9 < AUC < 1) and perfect diagnostic biomarker (AUC =1) [76]. Our analysis revealed that 23 out of the 25 miRNAs could be considered highly accurate (0.9 < AUC < 1) or better (AUC =1) in at least one of the cohorts they were present, except for miR-296-5p and miR-127-3p which were found to be moderately accurate in all the datasets they were present (Figure 7 and Supplementary Table 6).

Besides, the miRNAs miR-28-3p, miR-193a-5p, miR-299-5p, miR-320a, miR-320b, miR-324-3p, miR-326, miR-339-5p, miR-486-5p and miR-574-3p had perfect AUC values of diagnostic biomarker (AUC =1) in the TCGA dataset, and miR-125a-5p was considered perfect diagnostic tool (AUC =1) in the GEO GSE33125 dataset. Our results indicate that these 11 miRNAs could help to differentiate tumour from normal tissue with 100% sensibility and 100% specificity (Figure 7 and Supplementary Table 6). Thus, these deregulated miRNAs can be powerful diagnostic tools for an early CRC detection, an observation also corroborated by other groups [36, 56, 57, 77–79].

### Identification of potential prognostic biomarkers for colorectal cancer

**Figure 8:**
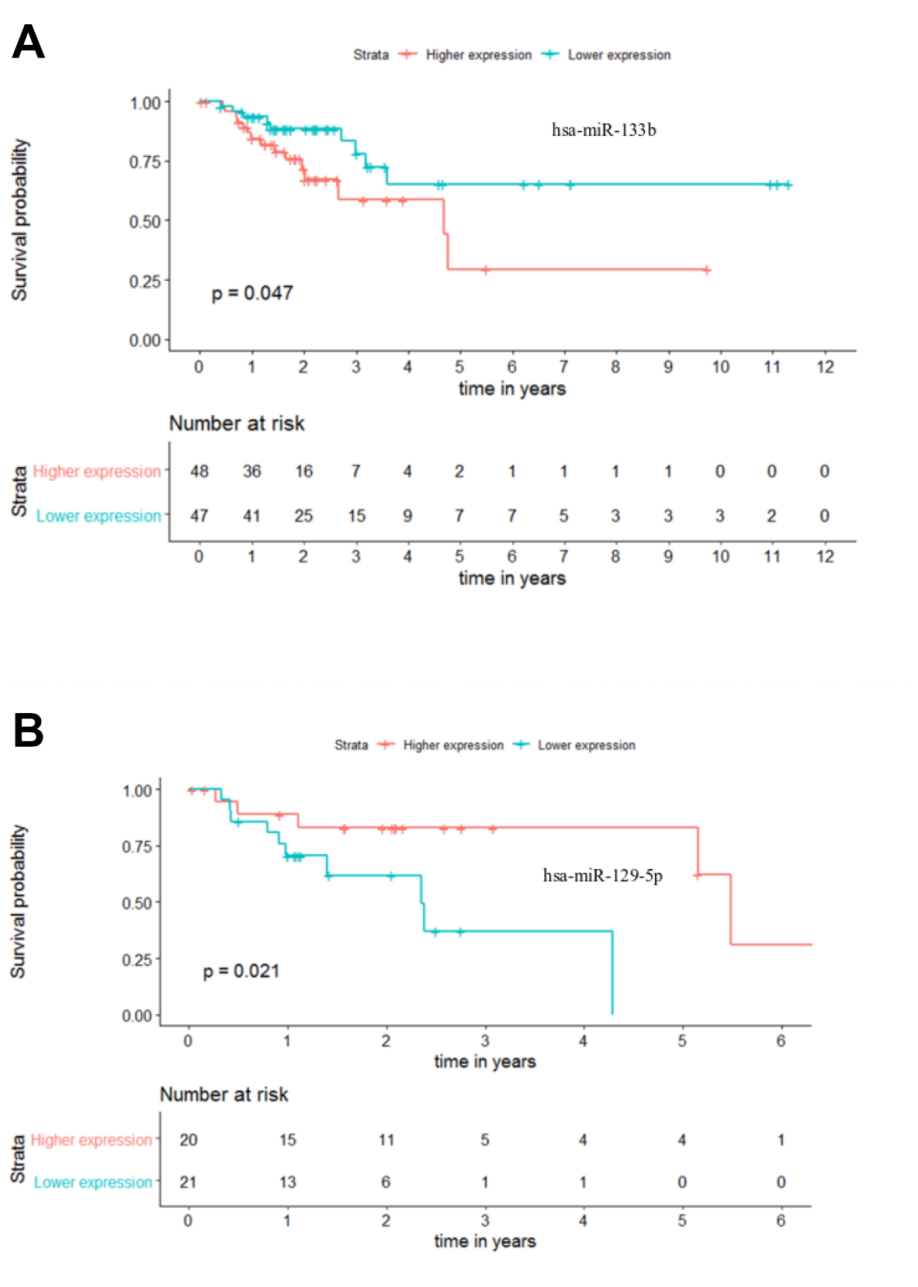
**Individual miRNA overall survival (OS) prognosis for stage III and stage IV patients.** (**A**) Stage III OS Kaplan-Meier curve for miR-133b (*p-value* = 0.047, Log rank; HR = 2.28) (**B**) Stage IV OS Kaplan-Meier curve for miR-129-5p (*p-value* = 0.021, Log rank; HR = 4.23). Time is represented in years. Higher (in red) and Lower (in blue) expression groups represent the patients with miRNA expression above and below miRNAs’ median expression, respectively. Censored data is represented by small plus signs in each group. The number of patients at risk for each group and per time point is shown in the table below each graph. HR, hazard ratio.

The current TNM staging system lacks the ability to accurately predict patient outcome, leading to suboptimal clinical management [17]. In this sense, we proceeded to investigate if the 25 differentially expressed miRNAs could differentiate patients within each stage of disease. To perform these analyses, Kaplan-Meier curves for Overall Survival (OS) and Recurrence Free Survival (RFS) were plotted using the median expression of each miRNA to form two distinct groups (“higher expression” group and “lower expression” group) with a minimum of 30 patients. Our analysis revealed that only miR-133b (*p-value* = 0.047) and miR-129-5p (*p-value* = 0.021), for stages III and IV respectively, could be potential solo OS biomarkers (Figure 8A, 8B). While a lower expression of miR-133b was indicative of a better outcome for patients in stage III, higher levels of miR-129-5p in stage IV patients seems to relate to extended overall survival (Figure 8A, 8B). This highlights the potential of miR-133b and miR-129-5p as individual OS biomarkers in CRC stages III and IV, respectively. On the other hand, none of the selected 25 miRNAs was able to perform as a good predictive biomarker for RFS. Nevertheless, it is highly unlikely that a single miRNA could provide a strong and precise prognostic tool. Instead, the combination of a group of miRNAs should be a more powerful clinical tool for patient management.

**Figure 9:**
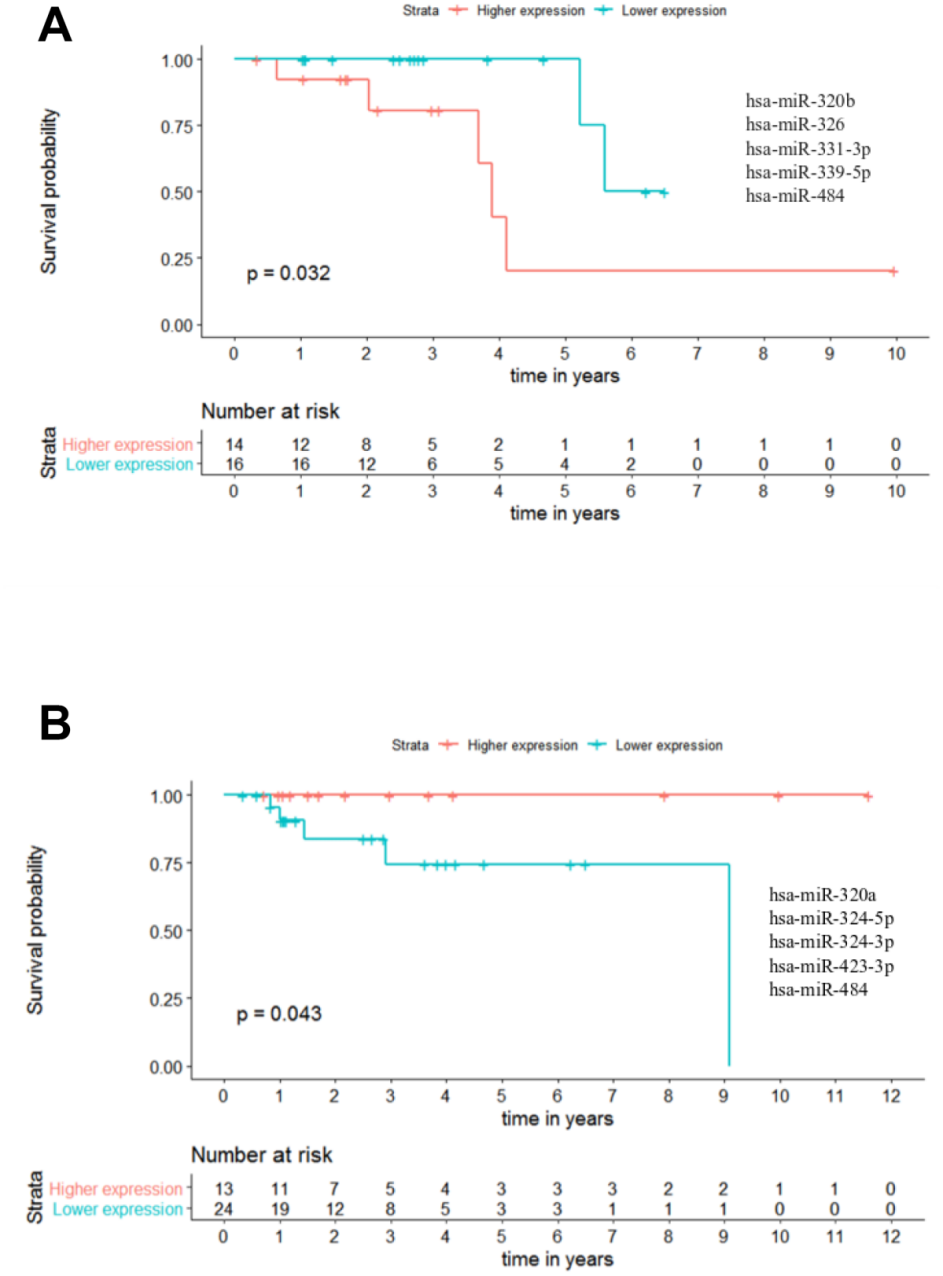
**Panels of 5 miRNAs for overall survival (OS) and recurrence free survival (RFS) prognosis of stage II patients.** (**A**) Stage II OS Kaplan-Meier curve based on miR-320b - miR-326 - miR-331-3p - miR-339-5p - miR-484 (*p-value* = 0.032, Log rank test; HR= 5.23) (**B**) Stage II recurrence free survival RFS Kaplan-Meier curve based on miR-320a - miR-324-5p - miR-324-3p - miR-423-3p - miR-484 (*p-value* = 0.043, Log rank test). Time is represented in years. Higher (in red) and Lower (in blue) expression groups represent the group of patients with miRNA expression above and below miRNAs median expression, respectively. Censored data is represented by small plus signs in each group. The number of patients at risk for each group and per time point is shown in the table below each graph. HR, hazard ratio.

In this context, we analysed the potential of the 25 miRNAs to distinguish patients with different outcomes in combinations of up to 5 miRNAs. A maximum of 5 miRNAs was set in order to assure a minimum of 30 patients when performing the analysis, as panels with more miRNAs reduced the number of patients to less than 30. These analyses were performed across all stages of CRC, however the results from stages I and IV were excluded due to the small number of patients (below 30). Nevertheless, with this approach we could identify one panel of 5 miRNAs able to predict the outcome of patients in stage II regarding OS. In this panel, composed by miR-320b - miR-326 - miR-331-3p - miR-339-5p - miR-484 (*p-value* = 0.032; hazard ratio (HR) = 5.23), the group of patients with lower miRNA expression had a better outcome with ~5 times more probability to be alive than the patients in the group with higher miRNAs expression (Figure 9A and Supplementary Table 7). Moreover, by combining less than 5 miRNAs we could also identify 27 panels of 4 miRNAs, 54 panels of 3 miRNAs and 6 panels of 2 miRNAs capable of predicting the overall survival of stage II patients in CRC (Supplementary Table 7).

Regarding RFS analysis, we could identify two panels of 5 miRNAs with prognostic value. The best panel was the one composed by miR-320a - miR-324-5p - miR-324-3p - miR-423-3p - miR-484 in which the group of patients with higher combined expression had a better outcome than the lower expression group (*p-value* = 0.043) (Figure 9B and Supplementary Table 8). Likewise, for the panel miR-320a - miR-320b - miR-324-3p - miR-423-3p - miR-484 the group of patients with higher miRNAs expression had a better outcome than the lower expression group (*p-value* = 0.049) (Supplementary Table 8). Additionally, we identified 1 panel of 4 miRNAs, 3 panels of 3 miRNAs and 3 panels of 2 miRNAs capable of predicting RFS for stage II patients in CRC (Supplementary Table 8).

**Figure 10:**
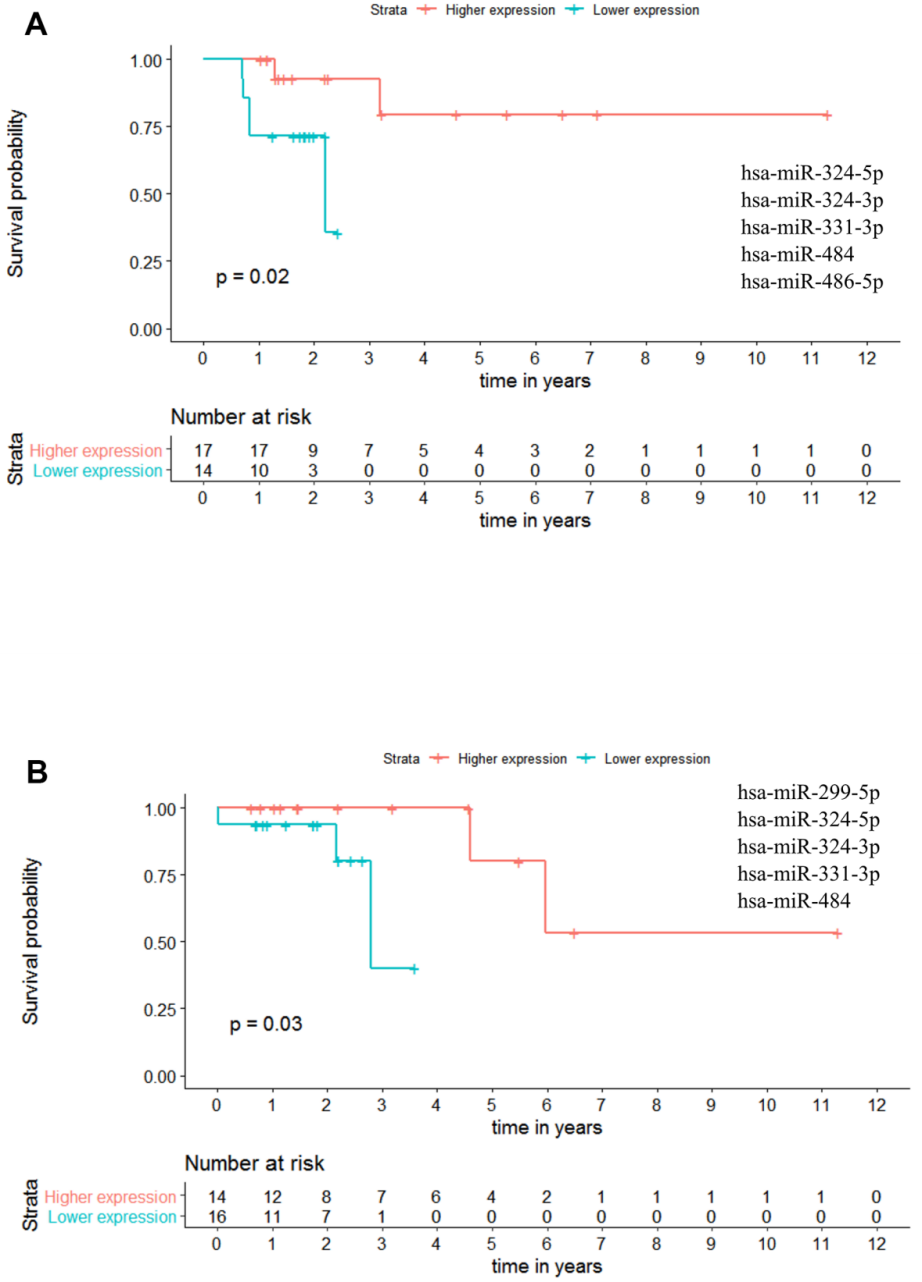
**Panels of 5 miRNAs for overall survival (OS) and recurrence free survival (RFS) prognosis of stage III patients.** (**A**) Stage III OS Kaplan-Meier curve based on miR-324-5p - miR-324-3p - miR-331-3p - miR-484 - miR-486-5p (*p-value* = 0.020, Log rank test; HR= 8.15). (**B**) Stage III recurrence free survival RFS Kaplan-Meier curve based on miR-299-5p - miR-324-5p - miR-324-3p - miR-331-3p - miR-484 (p = 0.030, Log rank test). Time is represented in years. Higher (in red) and Lower (in blue) expression groups represent the group of patients with miRNA expression above and below miRNAs median expression, respectively. Censored data is represented by small plus signs in each group. The number of patients at risk for each group and per time point is shown in the table below each graph. HR, hazard ratio.

Regarding Stage III patients, we have identified one panel with potential OS value: miR-324-5p - miR-324-3p - miR-331-3p - miR-484 - miR-486-5p (*p-value* = 0.020; HR = 8.150), indicating that at any given time, the patients with higher miRNAs expression had approximately 8 times more probability to be alive than the patients with lower expression levels (Figure 10A and Supplementary Table 9). Regarding RFS, one panel of 5 miRNAs showed potential prognostic value: miR-299-5p - miR-324-5p - miR-324-3p - miR-331-3p - miR-484 with a *p-value* = 0.030. With this panel of miRNAs, the patients presenting higher miRNAs expression have a better outcome than the patients with lower expression values (Figure 10B and Supplementary Table 10).

Moreover, for stage III patients we could identify a total of 65 remaining panels for OS (19 panels of 4 miRNAs, 31 panels of 3 miRNAs and 15 panels of 2 miRNAs; Supplementary Table 9) and 27 panels for RFS (10 panels of 4 miRNAs, 14 panels of 3 miRNAs and 3 panels of 2 miRNAs; Supplementary Table 10). Altogether these results further evidence the power of panels of miRNAs to predict patient outcome regarding both overall and recurrence free survivals.

### Validation analysis

**Figure 11:**
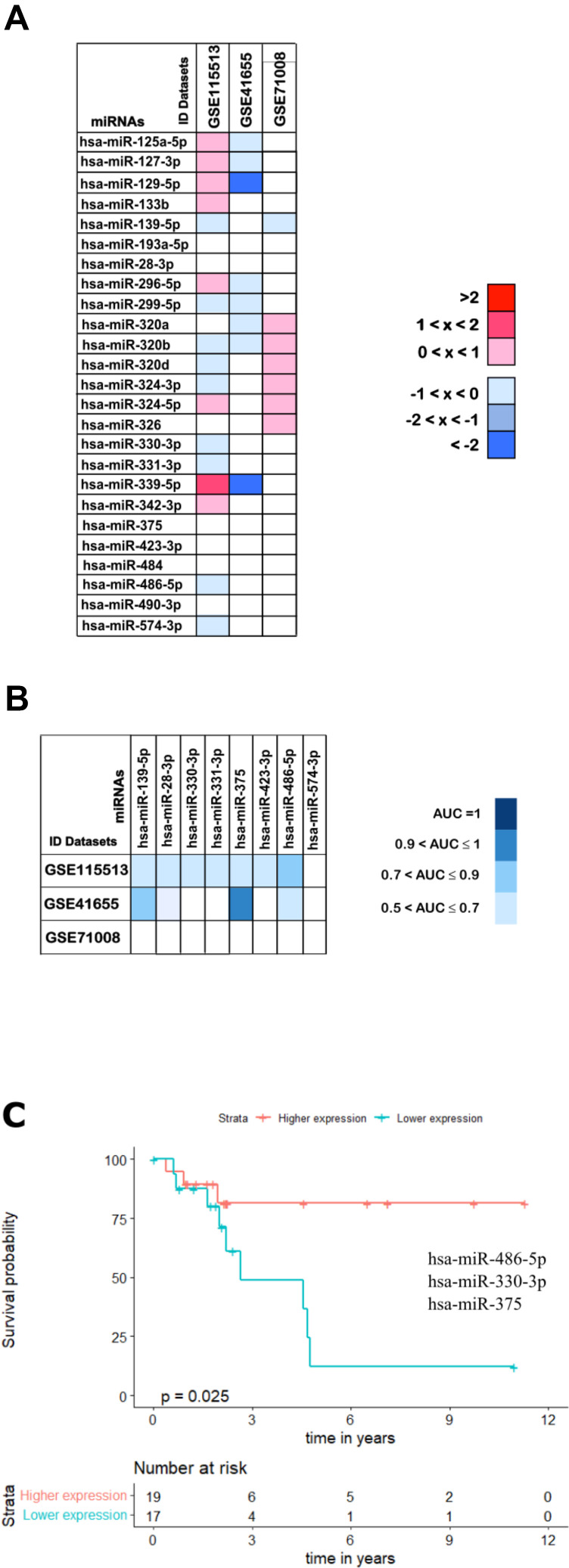
**Validation analysis for the 25 downregulated miRNAs in CRC.** (**A**) The log_2_(FC) values calculated for each dataset are reported with red scale boxes for upregulated miRNAs and blue scale boxes for the downregulated miRNAs. White boxes represent the inexistence of the miRNA on the dataset. (**B**) The miRNAs AUC values in each of the datasets GSE115513, GSE41655 and GSE71008 are reported as blue scale boxes. MiRNAs with AUC = 1 were considered perfect diagnostic biomarkers, 0.9 < AUC < 1 highly accurate, 0.7 < AUC ≤ 0.9 moderately accurate and 0.5 < AUC ≤ 0.7 less accurate [76]. (**C**) Stage III OS Kaplan-Meier curve based on miR-486-5p - miR-330-3p - miR-375 (*p-value* = 0.025, Log rank test; HR= 4.01). Time is represented in years. Higher (in red) and Lower (in blue) expression groups represent the group of patients with miRNA expression above and below miRNAs median expression, respectively. Censored data is represented by small plus signs in each group. The number of patients at risk for each group and per time point is shown in the table below each graph. HR, hazard ratio.

In order to validate our findings and further confirm the utility of the selected 25 miRNAs as potential non-invasive biomarkers for colorectal cancer we have inspected three additional and independent cohorts of CRC patients (validation cohorts). These included two cohorts with CRC tissue samples (GSE115513 and GSE41655 datasets) and one cohort with CRC plasma samples (GSE71008 dataset) plus the respective normal samples (Table 2). From these, the GSE41655 dataset showed the highest degree of similarity with the results described so far, with 23 out of the 25 miRNAs being downregulated in this cohort as well (Figure 11A). Although, since the total number of samples was relatively low (48 total samples) we have also analysed a larger cohort with a more significant number of cases (750 carcinomas and 649 normal mucosa samples). This analysis revealed that 12 out of the initial 25 miRNAs were also downregulated in the GSE115513 dataset (Figure 11A). Since miR-326 had no expression values available in this dataset for the normal mucosa samples we were unable to analyse its fold-change (Figure 11A).

**Table 2:** Detailed patient information for the three colorectal cancer datasets used to corroborate our analysis.

**Datasets**	**GSE115513**	**GSE41655**	**GSE71008**
Number of miRNAs available	2006	851	421
Patient nationality	USA	China	USA
Platform	Agilent-046064 Unrestricted Human miRNA V19.0 Microarray	Agilent-021827 Human miRNA Microarray	Illumina Genome Analyzer
Tissue type	Paired tumour and normal tissue	Tumour and normal tissue	Plasma microvesicles
**Number of samples**			
Tumour	750 (54%)	33 (69%)	100 (67%)*
Normal	649 (46%)	15 (31%)	50 (33%)*
**Age (mean ± SD^1^ years)**			
Tumour sample patients	65 ± 11	63 ± 11	55 ± 18
Normal sample patients	65 ± 11	48 ±14	54 ± 14
**Gender**			
Female	667 (48%)	16 (33%)	74 (49%)
Male	732 (52%)	32 (67%)	76 (51%)
**Tumour Site**			
Colon	792 (57%)	-	-
Rectum	607 (43%)	-	-

1 – standard deviation.

“-“– unspecified.

“*”– Tumour = cancerous patient samples; Normal = healthy individual samples.

Furthermore, and toward identifying miRNAs that could be used as non-invasive diagnostic and prognostic tools, we have investigated if our results could be extrapolated to a plasma context. Therefore, the expression profile of the 25 downregulated miRNAs was assessed in a cohort with plasma samples from CRC patients (GSE71008 dataset; Figure 11A). MiR-133b, miR-296-5p and miR-299-5p were not present in the GSE71008 dataset and therefore their regulation status was not evaluated. For the remaining 22 miRNAs, 12 were found to be downregulated and thus presenting the same expression profile as in the tumour tissue (Figure 11A).

Interestingly, comparing the outcomes from the three validation datasets we were able to identify 8 miRNAs commonly downregulated: miR-139-5p, miR-28-3, miR-330-3p, miR-331-3p, miR-375, miR-423-3p, miR-486-5p and miR-574-3p, further corroborating part of our results from the set of ‘discovery’ cohorts (Figure 11A).

Finally, we proceed to validate the diagnostic and prognostic ability of the 8 miRNAs commonly downregulated in all three validation cohorts. Regarding diagnostic ability, the 8 miRNAs did not present accuracy values as high as the ones obtained in the set of ‘discovery’ cohorts, with most of the miRNAs showing AUCs in the range of 0.5 and 0.7 in all three datasets and only miR-375 reaching an AUC above 0.9 in the GSE41655 dataset (Figure 11B and Supplementary Table 11).

When considering prognostic ability, none of the validation datasets provided information regarding the OS or RFS of patients. Therefore, to infer the prognostic potential of these 8 miRNAs we had to use the patient information available in the TCGA dataset. Importantly, we found that the panel of 2 miRNAs containing miR-486-5p - miR-375 (*p-value* = 0.032; HR = 2.890) and the panels of 3 miRNAs containing miR-486-5p - miR-330-3p - miR-375 (*p-value* = 0.025; HR = 4.010) or miR-486-5p - miR-331-3p - miR-375 (*p-value* = 0.031; HR = 3.911) could predict survival outcomes of stage III patients (Supplementary Table 12 and Figure 11C).

## DISCUSSION

MiRNAs are key regulators of diverse biological and physiological processes and thus their deregulation is strongly associated with pathological contexts [38, 39, 42]. The role of these small RNAs in cancer, including in CRC, has been one of the most studied over the years [45, 54, 80–82]. MiRNAs are stable, easily measurable, detectable in body fluids allowing for a non-invasive evaluation and, they are targetable molecules [41, 42, 83]. Due to their characteristics, miRNAs are promising biomarkers for both the detection and treatment of diseases, including CRC.

From the major hurdles in cancer management, late detection of malignancy, poor patient stratification and consequent suboptimal treatment are probably the ones that most impact on patient survival and recurrence of disease (excluding metastasis). Here we propose to identify miRNAs able to accurately distinguish malignant from healthy tissue and deliver panels of miRNAs as prognostic biomarkers for better patient stratification, particularly for CRC stages II and III. For that, we started by an integrated analysis using 4 colorectal cancer miRNA expression datasets (discovery cohorts) to identify miRNAs which expression was altered upon disease initiation and progression. Most miRNA expression changes in CRC seem to occur during normal mucosa to tumour transformation and are maintained throughout metastatic transition [15], which is similar to what we observed. We have unveiled 26 deregulated miRNAs in tumour tissue, exhibiting the same downregulation profile in at least 3 of the 4 datasets studied. The same regulation pattern was described in previous studies in multiple human cancers, including CRC, pancreatic, prostate and breast cancer [84]. An explanation for the massive miRNA downregulation in cancer was reported previously by Sun et al. 2016 and others. The authors found that ERK suppresses pre-miRNA export from the nucleus into the cytoplasm through phosphorylation of exportin-5 (EXPO5) [85]. Accordingly, in another study, pre-miRNAs are retained in the nucleus of cancer cell lines implying that the function of the nuclear-cytoplasmatic export machinery is compromised in tumour cells [86]. Even though, other mechanisms such as deletions or mutations, transcriptional and epigenetic regulation, seem to contribute to this phenomenon [87, 88].

We then hypothesize that the 26 altered miRNAs may regulate key genes in CRC further contributing to tumour progression. Also, miRNA function is known to be better understood through their target genes and regulatory networks [39]. Thus, we used MirTarBase to unveil the downstream targets of the miRNAs in study, since it relies on experimentally validated miRNA:target interactions [89]. We found that 25 out of the 26 miRNAs targeted a total of 173 genes previously described to be altered in CRC thus supporting our first analysis and the relevance of these deregulated miRNAs in colorectal cancer [90]. From the 312 miRNA:target interactions found, 104 corresponded to significant correlations between the expressions of the intervenient miRNA and target gene. Importantly, 22 out of the 25 miRNAs with interactions from the first analysis were maintained after the correlation analysis corroborating our approach. More than half of these statistically significant interactions were positive correlations, meaning that the expressions of miRNA and target gene evolve in the same direction. Although we should expect a decrease in the target levels upon an increase of the miRNA (or the other way around), it is well known that miRNAs are fine-tuning regulators of gene expression by primarily blocking protein translation rather than mRNA cleavage [20, 38, 39, 67, 69, 91]. Besides, the MirTarBase interactions were validated in a variety of backgrounds and not only in CRC contexts [20, 67, 69, 89, 91]. Therefore, future studies should confirm that the miRNA:target associations here identified also occur in colorectal cancer settings. Nevertheless, all miRNAs were found to intervene in signalling pathways usually deregulated in CRC such as the ones of WNT, TGFβ, TP53, PI3K-AKT, Ras and MAPK. Various studies have already demonstrated the key role of miRNAs in regulating these pathways [4, 20, 67, 69, 91–95] thus reinforcing our findings. Furthermore, Reid and colleagues showed that many miRNAs deregulated in CRC were computationally mapped to targets involved in pathways related to tumour progression [96]. Overall, these reports and our results suggest that miRNAs may contribute to the deregulation of important genes and cellular pathways that initiate and sustain the carcinogenic process. If this is the case, the miRNAs here identified should help detect precancerous and/or cancerous lesions and, in fact, we found they all have diagnostic value as 23 out of 25 are considered highly accurate or better biomarkers (AUC >0.9) in at least one of the datasets they were present. Among those, 11 miRNAs could differentiate tumour from normal tissue with 100% sensibility and 100% specificity. These same miRNAs had already been reported as potential biomarkers in CRC in several independent studies corroborating our findings [30–37]. Importantly, the potential biomarkers here identified surpass by far the sensibilities and specificities of the ones used nowadays in a clinical setting such as CEA and CA-19 [14, 15, 97]. Both proteins are found significantly increased in the serum of CRC patients although, usually in late stages of disease thus questioning their value for early detection of CRC [14, 15]. On the other hand, miRNAs have been considered promising non-invasive biomarkers for disease since the discovery that they can also be found in circulation [98–100]. In that sense, we propose that these 11 miRNAs which could accurately identify tumour tissue, might preserve their diagnostic biomarker status in blood samples and this should be addressed in future studies. Supporting our premise, the majority of the diagnostic miRNAs here described were already found circulating in blood [33, 99, 101–104] and in fact 4 out of these 11 miRNAs present the same expression profile in the plasma cohort here analysed.

Moreover, optimal clinical management of CRC regarding risk stratification of patients is still an unmet need. The current TNM staging system lacks the ability to accurately predict patient outcome, even in patients within the same stage, leading to suboptimal treatment administration [17, 19]. Interestingly, the majority of the 25 differentially expressed miRNAs had predictive value in panels with 2 to 5 miRNAs. Principally, these panels of miRNAs were able to predict the outcome of patients in stages II and III, the two disease stages for which risk stratification and therapeutic options are more challenging [17, 19]. Interestingly, some miRNAs were more associated with prognosis of either stage II or III, being part of a higher number of panels in a specific stage. For stage II OS we could evidence the role of miR-331-3p, miR-320b, miR-342-3p and miR-324-5p participating in an average of 15 (out of 87) panels. On the other hand, miR-324-3p, miR-375 and miR-486-5p participated in about 20 (out of 66) panels for stage III OS prognosis. These findings highlight some miRNAs that might specifically contribute to the prognosis of a specific CRC stage. In fact, all of them were previously described as tumour suppressor miRNAs by targeting genes that primarily control cell proliferation and/or apoptosis [105–109]. These reports are in agreement with the downregulation profile we observe for the same miRNAs in the present study. Additionally, the significant correlations for the miRNA:target interactions we have identified are reported by others as crucial for the function of these miRNAs. In CRC cell lines, miR-320b was found to target *MYC* to suppress cell proliferation [105] while miR-331-3p besides inhibiting proliferation additionally stimulates apoptosis by targeting *HER2* through activation of the PI3K/Akt and ERK1/2 pathways [106]. Inhibition of cell growth in cervical and gastric cancers by miR-331-3p was also reported via targeting *NRP2* and *E2F1* respectively [107, 108]. Interestingly, our analysis depicted several target genes belonging to the same signalling pathways but targeted by distinct miRNAs, suggesting that different miRNAs might work in synergy to activate or repress specific signalling cascades. Corroborating our findings, Ferretti and colleagues reported that miR-324-5p, miR-125b and miR-326 work together to activate Hedgehog signalling by targeting *GLI1* and Smoothened [109], the same targets we predicted with our analysis. Thus, we propose that in CRC the downregulation of these miRNAs might lead to high levels of Hedgehog downstream genes, contributing to tumour cell proliferation.

Moreover, miR-324-3p regulates *WNT2B*, one of the WNT ligands able to activate the WNT pathway and suppresses migration and invasion in nasopharyngeal carcinoma [110]. The interaction of miR-486-5p and components of insulin growth factor (IGF) signalling, is another example of regulations depicted in our analysis and reported in the literature. By targeting *IGF1*, *IGF1R* and *PIK3R1* this miRNA induces cell cycle arrest and reduces migration of lung cancer cells while acting as a tumour suppressor in a xenograft mouse model [111]. In a similar model for CRC, miR-375 could significantly reduce tumour growth *in vivo* and its pro-apoptotic role in colorectal cancer was associated with the targeting of *YAP1* [112]. These studies further corroborate our analysis and strengthen our findings, which highlight combinations of miRNAs that might help clinical decisions in the future. Two of these combinations, one for stage II and one for stage III, are composed of panels of 5 miRNAs as prognostic biomarkers for overall survival, sharing 2 miRNAs (miR-331-3p and miR-484). The targeting of *ZEB1* and *SMAD2* by miR-484 was predicted in our analysis and is reported to suppress proliferation and epithelial-mesenchymal transition in cervical cancer [113]. While for stage II the lower expression of the combined 5 miRNAs was indicative of better prognosis, for stage III the opposite was true. This pattern is maintained for most of the miRNA’s combinations regarding stages II and III OS. This suggests that the expression of each miRNA in cancer must be meticulously controlled throughout tumour progression since small changes in miRNAs levels seem to have a high impact on patient outcome. The strict transcriptional regulation of miRNAs as well as feedback loops altering the expression of some miRNAs has been reported in several contexts [39, 111, 112, 114–116].

Some of the miRNAs in study also showed a predictive value regarding recurrence-free survival. Of those, miR-320d, miR-324-3p, miR-324-5p and miR-331-3p were shared in more miRNAs panels (average of 9/28) for stage III RFS prognosis, supporting the validity of our findings. For each of II and III stages of disease, we report a panel of 5 miRNAs with the ability to predict patient outcome, where higher miRNA levels are indicative of better prognosis. Corroborating our data, the predictive value of these miRNAs was previously described in cancer [59–61, 117].

Notably, we were able to validate the results of about one third (8 miRNAs) out of the 25 downregulated miRNAs simultaneously in three new cohorts (validation cohorts) regarding both their regulation status and biomarker potential. One of the validation cohorts is composed by plasma samples further corroborating our results and highlighting the potential of these miRNAs as non-invasive CRC biomarkers. Interestingly, among these 8 miRNAs were miR-486-5p, miR-375 and miR-331-3p which had shown to provide great prognostic results in several miRNAs panels for stages II and III in the ‘discovery’ analysis. These results were further outlined when combinations with only the 8 miRNAs were used for prognostic analysis and still, we were able to generate 3 miRNAs panels able to predict survival in stage III CRC patients.

Here we highlight promising candidate biomarkers for diagnosis and prognosis of colorectal cancer. We focused on delivering panels of miRNAs able to stratify the risk of patients with stages II and III of CRC while evidencing miRNAs potential as non-invasive biomarkers. The combination of a group of biomarkers for cancer management is considered a reliable asset for the clinic [59–61] and has in fact became a thyroid cancer diagnostic commercial tool. Finally, we spot possible therapeutical targets for CRC since most of the downregulated miRNAs we have found act as tumour suppressors and manipulation of their levels should arrest cancer progression. MiRNA-based therapy is thus a promising avenue for future treatment options in cancer [41].

## MATERIALS AND METHODS

### Colorectal cancer miRNA expression data collection

MiRNA profiling data in CRC was collected from two repositories: The Cancer Genome Atlas (TCGA) database and the Gene Expression Omnibus DataSets portal (GEO DataSets).

TCGA CRC miRNA expression data was obtained from the miRNA mature strand expression RNAseq – IlluminaHiseq dataset for both TCGA Colon Cancer (COAD) and Rectal Cancer (READ;) cohorts (https://tcga.xenahubs.net) using the University of California, Santa Cruz cancer (UCSC) Xena Public Data Hubs (https://xena.ucsc.edu/pubichubs/) [118, 119].

GEO selection of the CRC datasets publicly available on NCBI (https://www.ncbi.nlm.nih.gov/geo/) was carried out by searching the terms in the advanced research tool “(microRNA expression) AND (Illumina) AND (colorectal cancer) AND "Homo sapiens"[porgn:__txid9606]” and “(microRNA expression) AND (colorectal cancer) AND "Homo sapiens"[porgn:__txid9606]”. Only the datasets containing the miRNA expression data for both colorectal cancer tissue samples and healthy tissue counterpart were included in our study. Exclusion criteria for the discovery cohorts were: i) datasets containing information only regarding cancer patients or pathological tissue samples; ii) datasets containing information on miRNAs expression levels for plasma samples only; iii) datasets that studied a set of specifically determined miRNAs. Four GEO datasets fulfil our criteria: GSE30454, GSE54088, GSE18392 and GSE33125. Solely GEO datasets containing miRNA expression obtained through Illumina platforms were used in the present work.

To identify the differently expressed miRNA in CRC, the data matrix of each of the 5 selected datasets was downloaded, transformed into a. csv file, and imported to R.

### Data processing – preparing data for analysis

The miRBase database (miRBase 21, http://www.mirbase.org/) was used to standardize miRNA nomenclature [120]. Missing data treatment was performed using a modified listwise case deletion, in which variable elimination was performed when more than half of the information for each miRNA was missing in either normal or colorectal cancer samples [121]. This technique represented a more conservative approach to listwise case deletion, as the last would result in the omission of an extensive number of data which could lead to having insufficient data to perform the analysis. This step allowed us to further reduce the noise in our results. Missing data treatment was proceeded by outlier detection, performed using the Box plot technique, and for each miRNA, outlier values in both tumour and normal groups were eliminated [122, 123]. After data processing, the GSE54088 dataset was removed from the analysis due to its low number of miRNAs (only 6) available for further analysis.

### Power consideration and statistical analysis

To assure that the miRNA expression values between normal and cancer patient samples were large enough to be considered statistically significant, a series of statistical tests were conducted. First, normal distribution assessment through Shapiro-Wilk test was performed. If miRNAs were normally distributed (*p-value* > 0.05) we applied a Wilcoxon-Mann-Whitney test, otherwise, a Levene’s test followed by a two-sample *t*-test was used to assess statistical differences between CRC and normal tissue samples [124–128]. A *p-value* of 0.05 was considered statistically significant in all tests. A False Discovery Rate (FDR) equal to or below 5% was considered statistically significant for both *t*-test and Mann-Whitney [129, 130].

### Identification of differently expressed miRNAs in colorectal cancer

MiRNA differential expression in CRC, for each dataset, was determined by the fold change (FC) values between colorectal cancer tumour and normal samples. After calculated, the FC values were then transformed in base-2 logarithm of FC (log_2_(FC)). The differently expressed miRNAs obtained for each dataset were then merged using a Venn diagram to identify miRNAs that presented statistical differences in at least 3 of our datasets.

### Identification of miRNAs target genes and functional analysis

MirTarBase version 7.0 was used to retrieve the target genes of the differentially expressed miRNAs until the 15^th^ of December 2019. MirTarBase is a manually collected dataset of microRNA-target interactions (MTIs) experimentally validated that holds more than four hundred and twenty thousand MTIs [89]. Only functional MTIs catalogued in *Homo sapiens* were considered in our analysis.

The miRNA-target genes reported to be involved in colorectal carcinoma development were obtained from the Colorectal Cancer Gene Database (CoReCG) (http://lms.snu.edu.in/corecg/) until the 22^nd^ of December of 2019 [131]. Visualization of the miRNA-target interaction analysis was obtained thought a Chord-diagram performed using the *circlize* R package.

Functional and pathway enrichment analyses were performed using Kyoto Encyclopedia of Genes and Genomes (KEEG) (https://www.genome.jp/kegg/) available at The Database for Annotation, Visualization and Integrated Discovery (DAVID) Bioinformatics Resources 6.8 (https://david.ncifcrf.gov/home.jsp). A *p-value* <0.05 was considered to indicate statistical significance.

### Gene expression analysis

Gene expression data was obtained from the colon and rectal gene expression RNAseq - IlluminaHiSeq dataset for both TCGA Colon Cancer (COAD) and Rectal Cancer (READ) cohorts as described above for the miRNAs. [118, 119].

Sample matching was performed to identify correspondent samples between the gene expression dataset and the miRNA expression dataset. Only matched samples were used in our analysis. FC values between colorectal cancer tumour and normal samples were log_2_ transformed. Visual display of the log_2_(FC) expression values was obtained using a circular bar plot.

### MiRNA-target gene interaction analysis

Gene expression normality was assessed using the Shapiro-Wilk test. Pearson’s correlation coefficient was utilized to estimate the correlation between miRNA expression levels and gene expression levels if both miRNA and gene followed normality, otherwise, the Spearman test was used [132]. A *p-value* of 0.05 was considered statistically significant, and correlations were classified according to Mukaka 2012 [132].

### Biomarker potential – diagnostic and prognostic value

Clinical data (time of overall survival and of recurrence-free survival) was retrieved for each stage of disease (I, II, III and IV) from the TCGA, the only database with clinical information available.

To access miRNAs diagnostic value, Receiver Operating Characteristic (ROC) curves were generated using the *roc* function found in the *pROC* package, and miRNA’s ability to behave as potential biomarkers were determined by the Area under the curve (AUC) and stratified according to the classification provided in Greiner et al., 2000 [76]. The AUC represents the overall performance of the classifier summarized over all possible cut-off values, with an ideal ROC curve hugging the top left corner. Thus, the higher the AUC the better the classifier [76, 133].

For prognosis value, we considered statistically significant a *p-value* <0.05 for the log-rank test when performing the Cox Proportional-Hazard regression Model [134, 135]. To perform this analysis, two distinct groups of patients were constructed based on the tumour samples miRNAs medians. Patients with miRNA expression values above the respective tumour samples miRNA median were allocated in the “Higher expression” group, while patients with miRNA expression values below the median were allocated in the “Lower median” group. The Cox Proportional-Hazard regressions were conducted separately for each of the four stages of disease (I, II, III, and IV) [136]. Hazard ratios (HR) which represented the instantaneous risk of dying from CRC over the course of life were obtained as the ratio of the hazard rates between the “Higher expression” and “Lower expression” groups. Wilcox regression Models were performed and graphical representations (Kaplan-Meier’s) were plotted [137].

### Validation cohorts and analysis

To validate our results, the methylation status, diagnostic and prognostic potential of the selected 25-downregulated miRNAs was further analysed in 3 additional datasets: two generated from platforms other than Illumina (GSE115513 and GSE41655 datasets) and a plasma dataset (GSE71008) generated from an Illumina platform. To obtain similar results we have transformed the raw data and expressed it as log2 values in order to obtain the same scale value as the previous datasets used in this work.

### Statistical software

Analyses were performed using R studio version 3.6.4 [138] and supplemented by the R packages: car, outliers, dplyr, pROC, ggplot2, survival, survminer, VennDiagram and miRBaseVersions.db.

## Supplementary Material

Supplementary Figure 1

Supplementary Table 1

Supplementary Table 2

Supplementary Table 3

Supplementary Table 4

Supplementary Table 5

Supplementary Tables 6-12
