# Correction for: Identification of colorectal cancer associated biomarkers: an integrated analysis of miRNA expression

**DOI:** 10.18632/aging.203866

**Published:** 2022-02-28

**Authors:** André Fonseca, Sara Ventura Ramalhete, André Mestre, Ricardo Pires das Neves, Ana Marreiros, Pedro Castelo-Branco, Vânia Palma Roberto

**Affiliations:** 1 Faculty of Medicine and Biomedical Sciences (FMCB), University of Algarve, Campus de Gambelas, Faro, 8005-139, Portugal; 2 Algarve Biomedical Center Research Institute (ABC-RI), Faro, 8005-139, Portugal; 3 CNC, Center for Neuroscience and Cell Biology, CIBB - Centre for Innovative Biomedicine and Biotechnology, University of Coimbra, Coimbra, 3004-517, Portugal; 4 IIIUC-Institute of Interdisciplinary Research, University of Coimbra, Coimbra, 3030-789, Portugal; 5 Champalimaud Research Program, Champalimaud Center for the Unknown, Lisbon, 1400-038, Portugal; 6 Centre of Marine Sciences (CCMAR), University of Algarve, Faro, 8005-139, Portugal

Original article: Aging. 2021; 13:21991–22029.  https://doi.org/10.18632/aging.203556

**This article has been corrected:** The authors recently found that published **Figure 11** was an unfinished draft of the final figure. They are now providing the finalized figure referred to in the article. This alteration does not affect the results or conclusions of this work.

New **Figure 11** is presented below.

**Figure 11:**
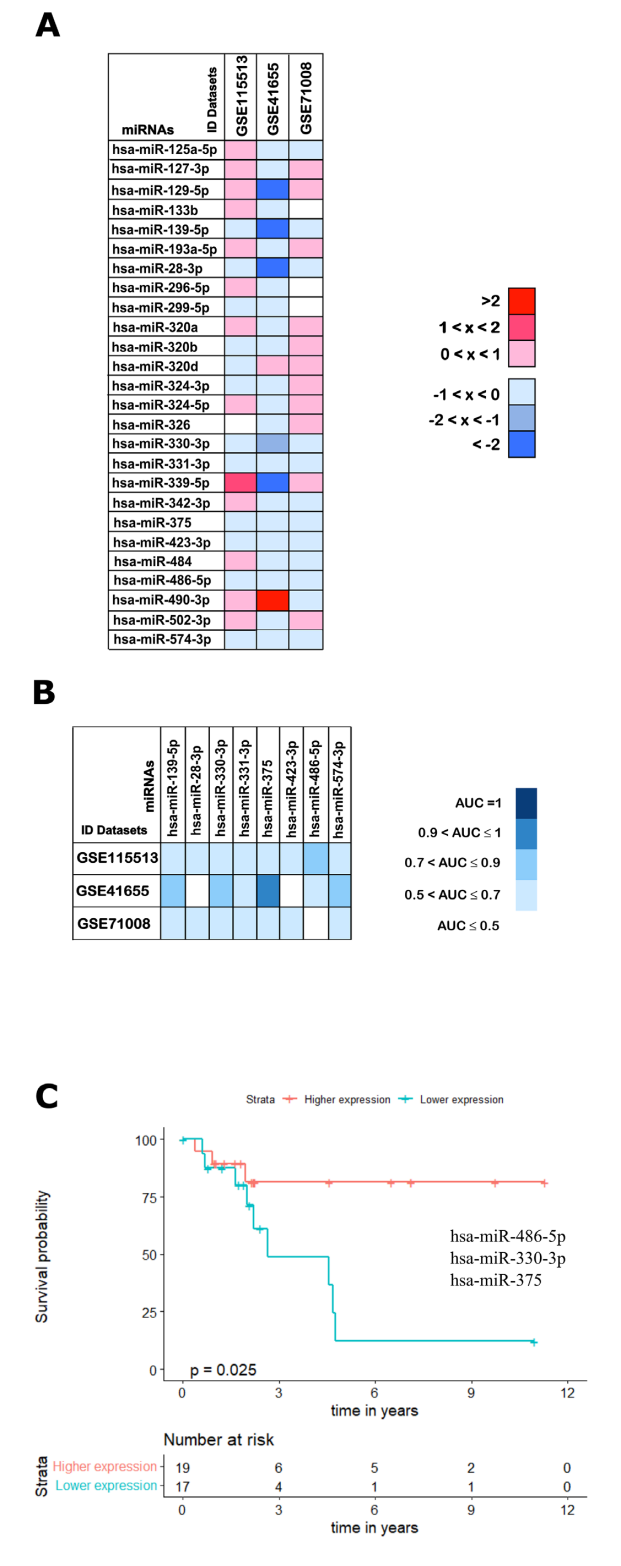
**Validation analysis for the 25 downregulated miRNAs in CRC.** (**A**) The log_2_(FC) values calculated for each dataset are reported with red scale boxes for upregulated miRNAs and blue scale boxes for the downregulated miRNAs. White boxes represent the inexistence of the miRNA on the dataset. (**B**) The miRNAs AUC values in each of the datasets GSE115513, GSE41655 and GSE71008 are reported as blue scale boxes. MiRNAs with AUC = 1 were considered perfect diagnostic biomarkers, 0.9 < AUC < 1 highly accurate, 0.7 < AUC ≤ 0.9 moderately accurate and 0.5 < AUC ≤ 0.7 less accurate [76]. (**C**) Stage III OS Kaplan-Meier curve based on miR-486-5p - miR-330-3p - miR-375 (*p-value* = 0.025, Log rank test; HR= 4.01). Time is represented in years. Higher (in red) and Lower (in blue) expression groups represent the group of patients with miRNA expression above and below miRNAs median expression, respectively. Censored data is represented by small plus signs in each group. The number of patients at risk for each group and per time point is shown in the table below each graph. HR, hazard ratio.

