# Supplementary material for: Identification of colorectal cancer associated biomarkers: an integrated analysis of miRNA expression
Source: Aging (Albany NY). 2021 Sep 21;13(18):21991–2029. doi: 10.18632/aging.203556 (PMC8507258; doi:10.18632/aging.203556)
Supplement: Supplementary Figure 1 [file aging-13-203556-s001.html]

edgebundleR
