# Supplementary material for: Identification of colorectal cancer associated biomarkers: an integrated analysis of miRNA expression
Source: Aging (Albany NY). 2021 Sep 21;13(18):21991–2029. doi: 10.18632/aging.203556 (PMC8507258; doi:10.18632/aging.203556)
Supplement: Supplementary Tables 6-12 [file aging-13-203556-s007.pdf]

SUPPLEMENTARY TABLES

Supplementary Table 6. Area under the ROC curves (AUC) for the 25 downregulated miRNAs according to each dataset.

MiRNA name	TCGA	GSE30454	GSE33125	GSE18392
hsa-miR-125a-5p	0.998	0.795	1.000	0.942
hsa-miR-127-3p	0.880	0.839		0.794
hsa-miR-129-5p	0.904	0.792		0.801
hsa-miR-133b	0.999	0.975		0.750
hsa-miR-139-5p	0.965	0.944	0.944	0.993
hsa-miR-193a-5p	1.000	0.793	0.986	
hsa-miR-28-3p	1.000	0.753	0.905	
hsa-miR-296-5p	0.875	0.859		0.882
hsa-miR-299-5p	1.000	0.842		0.764
hsa-miR-320a	1.000		0.864	0.929
hsa-miR-320b	1.000		0.864	0.929
hsa-miR-320d	0.695		0.864	0.929
hsa-miR-324-3p	1.000	0.739		0.940
hsa-miR-324-5p	0.934	0.754		0.841
hsa-miR-326	1.000	0.819		0.986
hsa-miR-330-3p	0.890		0.903	0.885
hsa-miR-331-3p	0.941	0.855		0.696
hsa-miR-339-5p	1.000	0.741		0.621
hsa-miR-342-3p	0.853	0.843	0.875	0.945
hsa-miR-375	0.917		0.972	0.970
hsa-miR-423-3p	0.990	0.677		0.849
hsa-miR-484	0.995	0.681	0.889	0.968
hsa-miR-486-5p	1.000	0.680	0.952	0.875
hsa-miR-490-3p	0.912	0.970		0.690
hsa-miR-574-3p	1.000	0.848		0.950

Supplementary Table 7. Stage II overall survival (OS) results for the panels of miRNAs containing combinations of 2, 3, 4 and 5 simultaneous miRNAs from the 25 selected miRNAs.

Stage 2 overal survival (OS) analysis				
**Combinations 2 by 2**				
**miRNA combinations**	**p-value**	**Number of individuals in the Higher expression group**	**Number of individuals in the Lower expression group**	**Hazard ratio**
hsa-miR-342-3p - hsa-miR-331-3p	0.000183	25	29	4.15E-10
hsa-miR-342-3p - hsa-miR-574-3p	0.0045	25	26	2.97E-10
hsa-miR-342-3p - hsa-miR-326	0.007241	26	29	0.132538444
hsa-miR-127-3p - hsa-miR-331-3p	0.020103	28	29	0.124275996
hsa-miR-127-3p - hsa-miR-342-3p	0.026718	26	27	0.187519247
hsa-miR-342-3p - hsa-miR-324-5p	0.040052	30	34	0.257492788
**Combinations 3 by 3**				
**miRNA combinations**	**p-value**	**Number of individuals in the Higher expression group**	**Number of individuals in the Lower expression group**	**Hazard ratio**
hsa- miR- 342- 3p - hsa- miR- 331- 3p - hsa- miR- 324- 5p	0.000371	18	23	1.91E-10
hsa- miR- 342- 3p - hsa- miR- 331- 3p - hsa- miR- 484	0.000644	17	23	2.50E-10
hsa- miR- 342- 3p - hsa- miR- 331- 3p - hsa- miR- 320b	0.000797	16	19	1.67E-10
hsa- miR- 342- 3p - hsa- miR- 331- 3p - hsa- miR- 324- 3p	0.002392	19	20	1.63E-10
hsa- miR- 342- 3p - hsa- miR- 326 - hsa- miR- 331- 3p	0.00311	19	17	2.73E-10
hsa- miR- 342- 3p - hsa- miR- 331- 3p - hsa- miR- 339- 5p	0.0036	15	18	2.02E-10
hsa- miR- 320a - hsa- miR- 342- 3p - hsa- miR- 331- 3p	0.004512	16	17	2.03E-10
hsa- miR- 330- 3p - hsa- miR- 342- 3p - hsa- miR- 331- 3p	0.004909	17	16	2.08E-10
hsa- miR- 342- 3p - hsa- miR- 326 - hsa- miR- 324- 5p	0.006234	14	19	0.082704817
hsa- miR- 127- 3p - hsa- miR- 331- 3p - hsa- miR- 320b	0.007548	15	18	5.07E-10
hsa- miR- 342- 3p - hsa- miR- 331- 3p - hsa- miR- 574- 3p	0.00761	14	19	1.53E-10
hsa- miR- 342- 3p - hsa- miR- 331- 3p - hsa- miR- 193a- 5p	0.008237	14	17	1.63E-10
hsa- miR- 127- 3p - hsa- miR- 331- 3p - hsa- miR- 320d	0.008371	16	14	6.19E-10
hsa- miR- 127- 3p - hsa- miR- 326 - hsa- miR- 320b	0.008834	15	17	3.02E-10
hsa- miR- 342- 3p - hsa- miR- 331- 3p - hsa- miR- 28- 3p	0.009528	13	17	1.79E-10
hsa- miR- 342- 3p - hsa- miR- 331- 3p - hsa- miR- 423- 3p	0.010063	16	19	1.83E-10
hsa- miR- 342- 3p - hsa- miR- 484 - hsa- miR- 574- 3p	0.011389	14	18	2.04E-10
hsa- miR- 342- 3p - hsa- miR- 324- 3p - hsa- miR- 486- 5p	0.012555	17	15	2.17E-10
hsa- miR- 342- 3p - hsa- miR- 324- 5p - hsa- miR- 574- 3p	0.012883	17	21	3.52E-10
hsa- miR- 331- 3p - hsa- miR- 299- 5p - hsa- miR- 320d	0.015158	14	18	5.15E-10
hsa- miR- 127- 3p - hsa- miR- 320a - hsa- miR- 331- 3p	0.015451	16	17	5.20E-10
hsa- miR- 125a- 5p - hsa- miR- 342- 3p - hsa- miR- 574- 3p	0.017208	17	16	3.72E-10
hsa- miR- 342- 3p - hsa- miR- 326 - hsa- miR- 320b	0.017705	17	17	0.104040078
hsa- miR- 127- 3p - hsa- miR- 574- 3p - hsa- miR- 320b	0.019981	16	19	4.28E-10
hsa- miR- 342- 3p - hsa- miR- 574- 3p - hsa- miR- 320b	0.021539	13	18	1.16E-10
hsa- miR- 125a- 5p - hsa- miR- 342- 3p - hsa- miR- 331- 3p	0.02189	14	17	1.18E-10
hsa- miR- 129- 5p - hsa- miR- 133b - hsa- miR- 574- 3p	0.025347	15	15	2.74E-10
hsa- miR- 326 - hsa- miR- 574- 3p - hsa- miR- 320b	0.027324	19	22	0.166390667
hsa- miR- 342- 3p - hsa- miR- 484 - hsa- miR- 28- 3p	0.027724	15	19	0.114500372
hsa- miR- 342- 3p - hsa- miR- 339- 5p - hsa- miR- 574- 3p	0.02844	17	17	3.33E-10
hsa- miR- 342- 3p - hsa- miR- 324- 3p - hsa- miR- 484	0.029009	20	21	0.116029538
hsa- miR- 342- 3p - hsa- miR- 324- 5p - hsa- miR- 324- 3p	0.029462	20	24	0.129069723
hsa- miR- 342- 3p - hsa- miR- 331- 3p - hsa- miR- 486- 5p	0.029685	16	15	1.41E-10
hsa- miR- 331- 3p - hsa- miR- 299- 5p - hsa- miR- 320b	0.030283	18	21	6.25E-10
hsa- miR- 326 - hsa- miR- 331- 3p - hsa- miR- 320b	0.03173	23	28	0.278381887
hsa- miR- 342- 3p - hsa- miR- 324- 3p - hsa- miR- 320b	0.033438	17	22	0.129436651
hsa- miR- 127- 3p - hsa- miR- 331- 3p - hsa- miR- 486- 5p	0.033689	16	16	0.132743995
hsa- miR- 342- 3p - hsa- miR- 326 - hsa- miR- 193a- 5p	0.034183	17	19	0.126647253
hsa- miR- 127- 3p - hsa- miR- 342- 3p - hsa- miR- 299- 5p	0.034542	19	22	0.141023701
hsa- miR- 331- 3p - hsa- miR- 484 - hsa- miR- 299- 5p	0.036181	23	20	6.93E-10
hsa- miR- 296- 5p - hsa- miR- 423- 3p - hsa- miR- 484	0.036411	16	16	1791989256
hsa- miR- 342- 3p - hsa- miR- 326 - hsa- miR- 324- 3p	0.03694	17	18	0.12921111
hsa- miR- 127- 3p - hsa- miR- 320a - hsa- miR- 324- 5p	0.037925	16	19	5.73E-10
hsa- miR- 127- 3p - hsa- miR- 574- 3p - hsa- miR- 193a- 5p	0.038784	19	22	6.70E-10
hsa- miR- 299- 5p - hsa- miR- 574- 3p - hsa- miR- 193a- 5p	0.039263	22	22	7.03E-10
hsa- miR- 127- 3p - hsa- miR- 331- 3p - hsa- miR- 484	0.042952	18	18	7.30E-10
hsa- miR- 127- 3p - hsa- miR- 324- 5p - hsa- miR- 320b	0.042952	17	18	7.30E-10
hsa- miR- 127- 3p - hsa- miR- 331- 3p - hsa- miR- 324- 5p	0.042963	18	19	0.150536555
hsa- miR- 127- 3p - hsa- miR- 330- 3p - hsa- miR- 331- 3p	0.045563	13	18	0.142419981
hsa- miR- 127- 3p - hsa- miR- 326 - hsa- miR- 193a- 5p	0.045818	23	23	6.06E-10

hsa- miR- 139- 5p - hsa- miR- 342- 3p - hsa- miR- 574- 3p	0.047428	20	18	1.20E-09
hsa- miR- 299- 5p - hsa- miR- 574- 3p - hsa- miR- 320b	0.04939	20	21	4.59E-10
hsa- miR- 330- 3p - hsa- miR- 342- 3p - hsa- miR- 326	0.049694	22	21	0.2024995
hsa- miR- 127- 3p - hsa- miR- 324- 5p - hsa- miR- 486- 5p	0.049795	15	16	0.142344939

Combinations 4 by 4

miRNA combinations	p-value	Number of individuals in the Higher expression group	Number of individuals in the Lower expression group	Hazard ratio
hsa-miR-342-3p - hsa-miR-331-3p - hsa-miR-324-5p - hsa-miR-320b	0.000535	12	18	1.50E-10
hsa-miR-342-3p - hsa-miR-331-3p - hsa-miR-324-5p - hsa-miR-484	0.001118	13	20	1.19E-10
hsa-miR-342-3p - hsa-miR-331-3p - hsa-miR-484 - hsa-miR-320b	0.001827	12	18	7.11E-11
hsa-miR-342-3p - hsa-miR-331-3p - hsa-miR-324-5p - hsa-miR-324-3p	0.00253	13	17	1.68E-10
hsa-miR-342-3p - hsa-miR-331-3p - hsa-miR-324-3p - hsa-miR-484	0.004245	14	16	1.14E-10
hsa-miR-320a - hsa-miR-342-3p - hsa-miR-331-3p - hsa-miR-484	0.005893	13	17	1.33E-10
hsa-miR-326 - hsa-miR-331-3p - hsa-miR-324-5p - hsa-miR-320b	0.021147	18	22	0.2287878
hsa-miR-326 - hsa-miR-331-3p - hsa-miR-574-3p - hsa-miR-320b	0.021749	14	19	0.161899927
hsa-miR-326 - hsa-miR-324-5p - hsa-miR-574-3p - hsa-miR-320b	0.023646	13	19	0.164324
hsa-miR-342-3p - hsa-miR-324-5p - hsa-miR-324-3p - hsa-miR-484	0.026451	16	20	0.113930394
hsa-miR-331-3p - hsa-miR-324-5p - hsa-miR-299-5p - hsa-miR-320b	0.028684	13	17	5.04E-10
hsa-miR-326 - hsa-miR-331-3p - hsa-miR-339-5p - hsa-miR-320b	0.029346	15	18	0.188073968
hsa-miR-127-3p - hsa-miR-299-5p - hsa-miR-193a-5p - hsa-miR-320b	0.031653	12	18	0.128504824
hsa-miR-127-3p - hsa-miR-324-5p - hsa-miR-484 - hsa-miR-299-5p	0.035638	16	17	7.99E-10
hsa-miR-331-3p - hsa-miR-299-5p - hsa-miR-193a-5p - hsa-miR-320b	0.035737	14	17	5.35E-10
hsa-miR-331-3p - hsa-miR-324-5p - hsa-miR-484 - hsa-miR-299-5p	0.035855	17	16	6.87E-10
hsa-miR-326 - hsa-miR-331-3p - hsa-miR-320b - hsa-miR-320d	0.03676	14	21	0.257376989
hsa-miR-127-3p - hsa-miR-330-3p - hsa-miR-299-5p - hsa-miR-193a-5p	0.037357	16	16	5.67E-10
hsa-miR-127-3p - hsa-miR-324-5p - hsa-miR-299-5p - hsa-miR-193a-5p	0.040035	15	18	7.07E-10
hsa-miR-127-3p - hsa-miR-299-5p - hsa-miR-574-3p - hsa-miR-320b	0.040135	15	17	4.06E-10
hsa-miR-331-3p - hsa-miR-484 - hsa-miR-299-5p - hsa-miR-320b	0.040575	15	15	5.89E-10
hsa-miR-127-3p - hsa-miR-326 - hsa-miR-299-5p - hsa-miR-193a-5p	0.040887	19	20	5.82E-10
hsa-miR-127-3p - hsa-miR-331-3p - hsa-miR-484 - hsa-miR-299-5p	0.04118	17	16	7.16E-10
hsa-miR-342-3p - hsa-miR-324-5p - hsa-miR-324-3p - hsa-miR-320b	0.041366	13	20	0.139955123
hsa-miR-127-3p - hsa-miR-299-5p - hsa-miR-574-3p - hsa-miR-193a-5p	0.043999	18	19	7.11E-10
hsa-miR-125a-5p - hsa-miR-324-5p - hsa-miR-574-3p - hsa-miR-320b	0.047546	13	18	0.198082863
hsa-miR-331-3p - hsa-miR-484 - hsa-miR-299-5p - hsa-miR-574-3p	0.048803	17	16	6.34E-10

Combinations 5 by 5

miRNA combinations	p-value	Number of individuals in the Higher expression group	Number of individuals in the Lower expression group	Hazard ratio
hsa-miR-326-hsa-miR-331-3p-hsa-miR-339-5p-hsa-miR-484-hsa-miR-320b	0.031802	14	16	0.191311093

Supplementary Table 8. Stage II recurrence-free survival (RFS) results for the panels of miRNAs containing combinations of 2, 3, 4 and 5 simultaneous miRNAs from the 25 selected miRNAs.

Stage 2 recurrence-free survival (RFS) analysis				
**Combinations 2 by 2**				
**miRNA combinations**	**p-value**	**Number of individuals in the Higher expression group**	**Number of individuals in the Lower expression group**	**Hazard ratio**
hsa-miR-129-5p - hsa-miR-423-3p	0.012472997	20	23	9.843039717
hsa-miR-129-5p - hsa-miR-486-5p	0.03761568	17	20	1223790672
hsa-miR-331-3p - hsa-miR-320d	0.039159223	28	30	0.331858185
**Combinations 3 by 3**				
**miRNA combinations**	**p-value**	**Number of individuals in the Higher expression group**	**Number of individuals in the Lower expression group**	**Hazard ratio**
hsa-miR-129-5p - hsa-miR-324-3p - hsa-miR-423-3p	0.012135384	16	14	2891271744
hsa-miR-129-5p - hsa-miR-324-5p - hsa-miR-423-3p	0.018478048	16	14	3747635084
hsa-miR-423-3p - hsa-miR-484 - hsa-miR-299-5p	0.042449644	17	17	4.611534615
**Combinations 4 by 4**				
**miRNA combinations**	**p-value**	**Number of individuals in the Higher expression group**	**Number of individuals in the Lower expression group**	**Hazard ratio**
hsa-miR-320a - hsa-miR-324-3p - hsa-miR-423-3p - hsa-miR-484	0.025165735	15	25	1495885403
**Combinations 5 by 5**				
**miRNA combinations**	**p-value**	**Number of individuals in the Higher expression group**	**Number of individuals in the Lower expression group**	**Hazard ratio**
hsa-miR-320a - hsa-miR-324-5p - hsa-miR-324-3p - hsa-miR-423-3p - hsa-miR-484	0.042737763	13	24	1516837907
hsa-miR-320a - hsa-miR-324-3p - hsa-miR-423-3p - hsa-miR-484 - hsa-miR-320b	0.049294563	14	22	1725069778

Supplementary Table 9. Stage III overall survival (OS) results for the panels of miRNAs containing combinations of 2, 3, 4 and 5 simultaneous miRNAs from the 25 selected miRNAs.

Stage 3 overal survival (OS) analysis				
**Combinations 2 by 2**				
**miRNA combinations**	**p-value**	**Number of individuals in the Higher expression group**	**Number of individuals in the Lower expression group**	**Hazard ratio**
hsa-miR-133b - hsa-miR-574-3p	0.012378	21	23	0.232222735
hsa-miR-375 - hsa-miR-324-3p	0.012638	29	31	4.407768211
hsa-miR-129-5p - hsa-miR-324-3p	0.014643	20	20	5.499440216
hsa-miR-129-5p - hsa-miR-486-5p	0.019913	25	25	5.066314309
hsa-miR-133b - hsa-miR-320b	0.021693	21	21	0.200765726
hsa-miR-129-5p - hsa-miR-375	0.021949	27	29	3.964313324
hsa-miR-127-3p - hsa-miR-375	0.028557	23	26	3.316305702
hsa-miR-127-3p - hsa-miR-486-5p	0.029109	27	28	2.967651866
hsa-miR-375 - hsa-miR-486-5p	0.031743	30	32	2.889364248
hsa-miR-324-3p - hsa-miR-486-5p	0.033261	30	31	2.924417356
hsa-miR-127-3p - hsa-miR-324-3p	0.034171	25	26	3.252558631
hsa-miR-133b - hsa-miR-423-3p	0.036065	16	14	0.141561917
hsa-miR-125a-5p - hsa-miR-133b	0.03707	29	26	0.343839238
hsa-miR-125a-5p - hsa-miR-574-3p	0.040105	25	27	0.373019785
hsa-miR-125a-5p - hsa-miR-320b	0.042205	26	27	0.288138938
**Combinations 3 by 3**				
**miRNA combinations**	**p-value**	**Number of individuals in the Higher expression group**	**Number of individuals in the Lower expression group**	**Hazard ratio**
hsa-miR-127-3p - hsa-miR-324-3p - hsa-miR-484	0.006047	15	16	7.109617001
hsa-miR-139-5p - hsa-miR-133b - hsa-miR-574-3p	0.00666	13	19	0.183225311
hsa-miR-129-5p - hsa-miR-375 - hsa-miR-330-3p	0.00815	15	15	10.62715282
hsa-miR-375 - hsa-miR-324-3p - hsa-miR-484	0.009241	19	25	9.673416356
hsa-miR-375 - hsa-miR-324-3p - hsa-miR-486-5p	0.010604	18	22	5.785941523
hsa-miR-375 - hsa-miR-324-3p - hsa-miR-299-5p	0.010849	17	20	9.195008842
hsa-miR-375 - hsa-miR-324-5p - hsa-miR-324-3p	0.015211	18	21	8.538590986
hsa-miR-375 - hsa-miR-326 - hsa-miR-324-3p	0.016561	19	22	8.384076984
hsa-miR-320a - hsa-miR-375 - hsa-miR-324-3p	0.019791	15	16	8.828596729
hsa-miR-324-5p - hsa-miR-324-3p - hsa-miR-486-5p	0.020048	23	22	4.372611975
hsa-miR-127-3p - hsa-miR-324-3p - hsa-miR-486-5p	0.020823	17	16	4.108012764
hsa-miR-324-3p - hsa-miR-484 - hsa-miR-486-5p	0.020911	21	25	4.149880583
hsa-miR-324-3p - hsa-miR-484 - hsa-miR-299-5p	0.02108	20	24	4.256428455
hsa-miR-324-3p - hsa-miR-486-5p - hsa-miR-299-5p	0.021618	17	20	4.987332351
hsa-miR-375 - hsa-miR-326 - hsa-miR-486-5p	0.022499	23	19	4.086629092
hsa-miR-375 - hsa-miR-331-3p - hsa-miR-484	0.023042	18	23	5.228028533
hsa-miR-127-3p - hsa-miR-324-3p - hsa-miR-299-5p	0.024471	20	19	5.035989942
hsa-miR-375 - hsa-miR-330-3p - hsa-miR-486-5p	0.025423	19	17	4.009611391
hsa-miR-331-3p - hsa-miR-324-3p - hsa-miR-299-5p	0.026531	22	26	3.557455347
hsa-miR-375 - hsa-miR-331-3p - hsa-miR-486-5p	0.031352	19	17	3.911348665
hsa-miR-375 - hsa-miR-324-3p - hsa-miR-339-5p	0.031628	16	18	5.059477829
hsa-miR-125a-5p - hsa-miR-574-3p - hsa-miR-320b	0.034116	14	17	0.255000527
hsa-miR-129-5p - hsa-miR-127-3p - hsa-miR-486-5p	0.035293	13	17	6.802202805
hsa-miR-127-3p - hsa-miR-484 - hsa-miR-486-5p	0.035369	14	17	3.820791631
hsa-miR-324-3p - hsa-miR-486-5p - hsa-miR-28-3p	0.036437	15	15	4.63958342
hsa-miR-375 - hsa-miR-324-3p - hsa-miR-28-3p	0.038203	16	16	7.255695673
hsa-miR-129-5p - hsa-miR-375 - hsa-miR-486-5p	0.041162	17	16	4.405278216
hsa-miR-331-3p - hsa-miR-486-5p - hsa-miR-299-5p	0.04183	19	20	3.542986862
hsa-miR-127-3p - hsa-miR-324-5p - hsa-miR-324-3p	0.042172	18	13	5.080609763
hsa-miR-129-5p - hsa-miR-375 - hsa-miR-326	0.042493	17	18	6.766177524
hsa-miR-330-3p - hsa-miR-324-3p - hsa-miR-299-5p	0.046184	18	21	4.411783414
**Combinations 4 by 4**				
**miRNA combinations**	**p-value**	**Number of individuals in the Higher expression group**	**Number of individuals in the Lower expression group**	**Hazard ratio**
hsa.miR.375-hsa.miR.324.3p-hsa.miR.484-hsa.miR.486.5p	0.003367	12	18	1846827734
hsa.miR.375-hsa.miR.324.3p-hsa.miR.484-hsa.miR.299.5p	0.008283	11	19	1378705155

hsa.miR.375-hsa.miR.331.3p-hsa.miR.324.3p-hsa.miR.484	0.008617	14	21	10.24584142
hsa.miR.331.3p-hsa.miR.324.3p-hsa.miR.484-hsa.miR.299.5p	0.008625	17	21	5.463558923
hsa.miR.331.3p-hsa.miR.324.3p-hsa.miR.486.5p-hsa.miR.299.5p	0.00879	15	16	6.031759481
hsa.miR.375-hsa.miR.326-hsa.miR.324.3p-hsa.miR.484	0.009778	13	18	1596889310
hsa.miR.127.3p-hsa.miR.331.3p-hsa.miR.324.3p-hsa.miR.299.5p	0.013565	15	16	5.858868629
hsa.miR.331.3p-hsa.miR.484-hsa.miR.486.5p-hsa.miR.299.5p	0.014746	14	17	5.734555866
hsa.miR.331.3p-hsa.miR.324.3p-hsa.miR.484-hsa.miR.486.5p	0.015847	19	19	4.546265704
hsa.miR.330.3p-hsa.miR.331.3p-hsa.miR.324.3p-hsa.miR.299.5p	0.019236	15	18	5.80803277
hsa.miR.324.3p-hsa.miR.484-hsa.miR.299.5p-hsa.miR.320b	0.024586	14	16	5.804163282
hsa.miR.375-hsa.miR.324.5p-hsa.miR.324.3p-hsa.miR.484	0.024595	14	17	1639582946
hsa.miR.375-hsa.miR.324.3p-hsa.miR.339.5p-hsa.miR.484	0.024757	13	17	8.740073882
hsa.miR.330.3p-hsa.miR.324.3p-hsa.miR.484-hsa.miR.299.5p	0.028156	13	17	7.652459638
hsa.miR.375-hsa.miR.324.3p-hsa.miR.484-hsa.miR.320b	0.031191	13	17	7.651464677
hsa.miR.375-hsa.miR.326-hsa.miR.331.3p-hsa.miR.324.3p	0.034775	13	18	7.145246862
hsa.miR.330.3p-hsa.miR.331.3p-hsa.miR.484-hsa.miR.486.5p	0.039314	16	14	4.011014862
hsa.miR.324.3p-hsa.miR.339.5p-hsa.miR.484-hsa.miR.299.5p	0.039558	15	15	5.098768587
hsa.miR.324.5p-hsa.miR.324.3p-hsa.miR.484-hsa.miR.486.5p	0.041095	18	17	5.134269181

Combinations 5 by 5

miRNA combinations	p-value	Number of individuals in the Higher expression group	Number of individuals in the Lower expression group	Hazard ratio
hsa-miR-331-3p - hsa-miR-324-5p - hsa-miR-324-3p - hsa-miR-484 - hsa-miR-486-5p	0.02038	17	14	8.81519013

Supplementary Table 10. Stage III recurrence-free Survival (RFS) results for the panels of miRNAs containing combinations of 2, 3, 4 and 5 simultaneous miRNAs from the 25 selected miRNAs.

Stage 3 recurence-free surival (RFS) analysis				
**Combinations 2 by 2**				
**miRNA combinations**	***p-value***	**Number of individuals in the Higher expression group**	**Number of individuals in the Lower expression group**	**Hazard ratio**
hsa-miR-127-3p - hsa-miR-320d	0.006346	19	24	1753902556
hsa-miR-324-3p - hsa-miR-320d	0.012169	21	21	5.7592911
hsa-miR-342-3p - hsa-miR-320d	0.046197	18	17	6.702539172
**Combinations 3 by 3**				
**miRNA combinations**	***p-value***	**Number of individuals in the Higher expression group**	**Number of individuals in the Lower expression group**	**Hazard ratio**
hsa-miR-127-3p - hsa-miR-326 - hsa-miR-320d	0.002417	16	15	2807731991
hsa-miR-127-3p - hsa-miR-330-3p - hsa-miR-320d	0.002723	12	19	2489265019
hsa-miR-326 - hsa-miR-299-5p - hsa-miR-320d	0.012646	15	15	3478257329
hsa-miR-330-3p - hsa-miR-299-5p - hsa-miR-320d	0.013823	16	16	10.5674672
hsa-miR-127-3p - hsa-miR-299-5p - hsa-miR-320d	0.016057	14	16	1933160272
hsa-miR-331-3p - hsa-miR-324-3p - hsa-miR-320d	0.019325	17	19	5.644369439
hsa-miR-296-5p - hsa-miR-486-5p - hsa-miR-574-3p	0.026561	13	17	3.32E-10
hsa-miR-125a-5p - hsa-miR-324-3p - hsa-miR-484	0.028803	15	17	2033531460
hsa-miR-127-3p - hsa-miR-28-3p - hsa-miR-320d	0.029091	13	18	1571292250
hsa-miR-125a-5p - hsa-miR-331-3p - hsa-miR-484	0.033347	14	16	8.256186753
hsa-miR-127-3p - hsa-miR-331-3p - hsa-miR-324-3p	0.035378	16	18	3.974019274
hsa-miR-375 - hsa-miR-324-5p - hsa-miR-339-5p	0.041514	20	20	0.151915197
hsa-miR-375 - hsa-miR-339-5p - hsa-miR-423-3p	0.043281	19	22	0.313552875
hsa-miR-375 - hsa-miR-423-3p - hsa-miR-320b	0.045428	17	18	0.269099457
**Combinations 4 by 4**				
**miRNA combinations**	***p-value***	**Number of individuals in the Higher expression group**	**Number of individuals in the Lower expression group**	**Hazard ratio**
hsa-miR-331-3p- hsa-miR-324-3p- hsa-miR-484- hsa-miR-299-5p	0.013791	15	19	10.59870794
hsa-miR-331-3p- hsa-miR-324-3p- hsa-miR-339-5p- hsa-miR-484	0.013997	16	17	1736443836
hsa-miR-375- hsa-miR-324-5p- hsa-miR-339-5p- hsa-miR-423-3p	0.016913	15	16	0.117756367
hsa-miR-324-5p- hsa-miR-423-3p- hsa-miR-574-3p- hsa-miR-320b	0.021091	17	13	5.59E-10
hsa-miR-324-5p- hsa-miR-339-5p- hsa-miR-423-3p- hsa-miR-574-3p	0.024983	16	14	4.64E-10
hsa-miR-324-5p- hsa-miR-324-3p- hsa-miR-484- hsa-miR-299-5p	0.027121	16	16	1244231980
hsa-miR-324-5p- hsa-miR-339-5p- hsa-miR-423-3p- hsa-miR-486-5p	0.028999	16	17	0.132653626
hsa-miR-320a- hsa-miR-331-3p- hsa-miR-324-3p- hsa-miR-484	0.037118	14	16	9.067631054
hsa-miR-324-5p- hsa-miR-423-3p- hsa-miR-486-5p- hsa-miR-320b	0.038805	15	15	0.139366947
hsa-miR-331-3p- hsa-miR-484- hsa-miR-299-5p- hsa-miR-574-3p	0.039364	14	17	1735697193
**Combinations 5 by 5**				
**miRNA combinations**	***p-value***	**Number of individuals in the Higher expression group**	**Number of individuals in the Lower expression group**	**Hazard ratio**
hsa-miR-331-3p - hsa-miR-324-5p - hsa-miR-324-3p - hsa-miR-484 - hsa-miR-299-5p	0.029939	14	16	1202659728

Supplementary Table 11. Area under the ROC curves (AUC) for the 25 downregulated miRNAs according to each new dataset.

MIRNA name	GSE115513	GSE41655	GSE71008
hsa-miR-125a-5p	0.537	0.541	0.7017
hsa-miR-127-3p	0.603	0.755	0.5232
hsa-miR-129-5p	0.512	0.546	0.5568
hsa-miR-133b	0.500	0.990	
hsa-miR-139-5p	0.602	0.818	0.6873
hsa-miR-193a-5p	0.498	0.748	0.5599
hsa-miR-28-3p	0.511	0.499	0.6416
hsa-miR-296-5p	0.516	0.589	
hsa-miR-299-5p	0.688	0.567	
hsa-miR-320a	0.533	0.688	0.5922
hsa-miR-320b	0.555	0.891	0.5315
hsa-miR-320d	0.537	0.546	0.5737
hsa-miR-324-3p	0.563	0.791	0.5714
hsa-miR-324-5p	0.583	0.796	0.5346
hsa-miR-326		0.539	0.5086
hsa-miR-330-3p	0.665	0.792	0.5769
hsa-miR-331-3p	0.577	0.560	0.5539
hsa-miR-339-5p	0.787	0.519	0.5094
hsa-miR-342-3p	0.540	0.884	0.5191
hsa-miR-375	0.568	0.923	0.5238
hsa-miR-423-3p	0.623	0.471	0.6176
hsa-miR-484	0.531	0.835	0.5273
hsa-miR-486-5p	0.737	0.682	0.4702
hsa-miR-490-3p	0.778	0.422	0.545
hsa-miR-574-3p	0.520	0.710	0.5996

Supplementary Table 12. Stage III overall survival (OS) results for the panels of miRNAs containing combinations of 2 and 3 simultaneous miRNAs from the selected 8 miRNAs.

Stage 3 overal survival (OS) analysis				
**Combinations 2 by 2**				
**miRNA combinations**	***p-value***	**Number of individuals in the Higher expression group**	**Number of individuals in the Lower expression group**	**Hazard ratio**
hsa-miR-486-5p - hsa-miR-375	0.031743	30	32	2.889364
**Combinations 3 by 3**				
**miRNA combinations**	***p-value***	**Number of individuals in the Higher expression group**	**Number of individuals in the Lower expression group**	**Hazard ratio**
hsa-miR-486-5p - hsa-miR-330-3p - hsa-miR-375	0.025423	19	17	4.009611
hsa-miR-486-5p - hsa-miR-331-3p - hsa-miR-375	0.031352	19	17	3.911349
